# CpG content in the Zika virus genome affects infection phenotypes in the adult brain and fetal lymph nodes

**DOI:** 10.3389/fimmu.2022.943481

**Published:** 2022-08-02

**Authors:** Daniel Udenze, Ivan Trus, Nathalie Berube, Uladzimir Karniychuk

**Affiliations:** ^1^ Vaccine and Infectious Disease Organization (VIDO), University of Saskatchewan, Saskatoon, SK, Canada; ^2^ School of Public Health, University of Saskatchewan, Saskatoon, SK, Canada; ^3^ Dioscuri Centre for RNA-Protein Interactions in Human Health and Disease, International Institute of Molecular and Cell Biology, Warsaw, Poland; ^4^ Department of Veterinary Microbiology, Western College of Veterinary Medicine, University of Saskatchewan, Saskatoon, SK, Canada

**Keywords:** CpG, CpG recoded virus, flavivirus, Zika virus, vaccine, viral evolution, neurotropic, pregnancy

## Abstract

Increasing the number of CpG dinucleotides in RNA viral genomes, while preserving the original amino acid composition, leads to impaired infection which does not cause disease. Beneficially, impaired infection evokes antiviral host immune responses providing a cutting-edge vaccine approach. For example, we previously showed that CpG-enriched Zika virus variants cause attenuated infection phenotypes and protect against lethal challenge in mice. While CpG recoding is an emerging and promising vaccine approach, little is known about infection phenotypes caused by recoded viruses *in vivo*, particularly in non-rodent species. Here, we used well-established mouse and porcine models to study infection phenotypes of the CpG-enriched neurotropic and congenital virus—Zika virus, directly in the target tissues—the brain and placenta. Specifically, we used the uttermost challenge and directly injected mice intracerebrally to compare infection phenotypes caused by wild-type and two CpG-recoded Zika variants and model the scenario where vaccine strains breach the blood-brain barrier. Also, we directly injected porcine fetuses to compare *in utero* infection phenotypes and model the scenario where recoded vaccine strains breach the placental barrier. While overall infection kinetics were comparable between wild-type and recoded virus variants, we found convergent phenotypical differences characterized by reduced pathology in the mouse brain and reduced replication of CpG-enriched variants in fetal lymph nodes. Next, using next-generation sequencing for the whole virus genome, we compared the stability of *de novo* introduced CpG dinucleotides during prolonged virus infection in the brain and placenta. Most *de novo* introduced CpG dinucleotides were preserved in sequences of recoded Zika viruses showing the stability of vaccine variants. Altogether, our study emphasized further directions to fine-tune the CpG recoding vaccine approach for better safety and can inform future immunization strategies.

## Introduction

The number of cytosine-phosphate-guanine (CpG) dinucleotides is underrepresented in vertebrates and most vertebrate RNA viruses (1, 2). Targeted increase of CpG dinucleotides in an RNA viral genome, while retaining the amino acid composition of encoded proteins, impairs infection *in vitro* and *in vivo* (3–9) providing a promising approach for live modified vaccines. In contrast to traditional live vaccines where few substitutions induce virus attenuation, CpG recoding is based on the cumulative effect of many nucleotide mutations resulting in hundreds of additional CpG dinucleotides that minimize reversion to the virulent state. Also, CpG recoding utilizes *de novo* gene synthesis and reverse genetics and may become an adaptable vaccine approach for rapid responses to emerging viruses. Recoded vaccine candidates with the increased CpG content encode viral proteins with the same amino acid sequence as the wild-type prototype inducing similar immune responses. For example, the CpG-recoded influenza virus caused attenuated infection in mice with reduced clinical severity and sterilizing immunity to lethal challenge (3). Our CpG-recoded Zika virus vaccine candidates also showed considerably reduced clinical disease and mortality in neonatal mice and safety in pregnant mice. Immunization with CpG-recoded Zika virus variants induced cellular and humoral immune responses comparable to responses induced by the wild-type variant and evoked complete protection against lethal heterologous challenge (10).

While CpG recoding is an emerging and promising vaccine approach, little is known about infection phenotypes caused by recoded viruses *in vivo*. Attenuated infection phenotypes caused by recoded vaccine candidates may depend on the expression of cellular components targeting viral CpG dinucleotides (8, 10–15). For example, based on analyses of archived gene expression data in different tissues, it has been suggested that central nervous tissues express low levels of the Zinc finger antiviral protein (ZAP)—the major known factor that specifically binds to the CpG-enriched regions of the virus genomic RNA (11). And CpG-dependent attenuation of infection caused by neurotropic viruses could be impaired in nervous tissues. In this context, comparative *in vivo* studies in different tissues will add to basic understanding and applied knowledge of the emerging CpG-recoding vaccine approach.

Here, we used the uttermost challenge and directly injected mice intracerebrally to compare infection phenotypes caused by wild-type and two CpG-recoded Zika virus variants and model the scenario where vaccine strains breach the blood-brain barrier. Also, we directly injected fetuses to compare *in utero* infection phenotypes and model the scenario where recoded vaccine strains breach the placental barrier.

## Materials and methods

### Design and recovery of CpG-recoded Zika virus variants

*In silico* recoding and recovery of CpG-modified Zika virus variants were previously described (10). We used the MUTATE SEQUENCES program in the SSE 1.3 software package (16) to modify the sequence of the contemporary Asian ZIKV H/PF/2013 strain [GenBank: KJ776791.2] (17). We generated a wild-type (WT) variant and variants with increased CpG numbers in regions encoding envelope (E)—E+102CpG, and E + nonstructural 1 (NS1) protein—E/NS1+176CpG (**Table 1**). In comparison to the WT variant, the E+102CpG variant contained 102 additional CpG dinucleotides in the genomic region encoding the E protein. The E/NS1+176CpG variant contained additional 102 and 74 CpG dinucleotides in the genomic regions encoding the E and NS1 proteins. Introduced nucleotide mutations did not alter the translated viral proteins. We also normalized frequencies of UpA dinucleotides in recoded Zika virus variants to the initial level. Recoded variants showed a modest reduction in codon pair bias scores in the E and NS1 genomic regions or minimal changes in the complete open reading frame (ORF) (10).

**Table 1:** Zika virus variants used in the study.

Genomic region	Zika variant	Number of CpG dinucleotides
E	Wild-type	35
	E+102CpG	137
	E/NS1+176CpG	137
NS1	Wild-type	25
	E+102CpG	25
	E/NS1+176CpG	99
ORF	Wild-type	316
	E+102CpG	418
	E/NS1+176CpG	492

To recover Zika virus variants, we used Infectious Subgenomic Amplicons (ISA) (17–19) as previously described (10). Recoded fragments were *de novo* synthesized (GenScript), amplified with high fidelity PCR (Invitrogen) and transfected into C6/36 Aedes albopictus mosquito cells (ATCC #CRL-1660) at +37°C for 12 h, and incubated for 7 days at +28°C (18). Media from virus-negative C6/36 cells was used as a control for transfection. After passaging twice in C6/36 cells, cell culture media containing Zika virus was centrifuged (12000 g, 20 min, +4°C), and frozen (-80°C) for subsequent animal experiments. Viral titers were quantified in triplicates in VERO cells with the endpoint dilution assay described below. All recovered Zika virus variants showed stability of *de novo* introduced CpG dinucleotides after ten passages in VERO cells (10). All virus stocks and cell cultures were free of mycoplasma contaminations as confirmed with the LookOut ^®^ Mycoplasma PCR Detection Kit (Sigma).

### Animal experiments

Specific pathogen-free eight- to ten-week-old BALB/c mice were purchased from Charles River Laboratories. Animals were housed at the Vaccine and Infectious Disease Organization (VIDO) biosafety level 2 mouse facility. To compare local brain-specific infection phenotypes caused by different Zika virus variants, BALB/c male and female mice (eighteen animals per group) were inoculated intracerebrally (IC) with the virus (10^3^ TCID_50_ in 30 µl DPBS) as we and others previously described (10, 20, 21). Control mice were inoculated with the same volume of virus-free media. Clinical signs—appearance, bodyweights, neurological signs—were monitored daily as previously described (10). Mice were euthanized at 7, 14, and 21 days after inoculation to sample blood, brain, and spleen for virus load and antibody response quantification, and next-generation sequencing (NGS).

Four pregnancy-synchronized Landrace-cross pigs were purchased from a high-health status herd free from porcine reproductive and respiratory syndrome virus (PRRSV) and porcine parvovirus (PPV) which can cause fetal infection. Pregnant pigs were randomly assigned into Control, E+102CpG, E/NS1+176CpG and WT groups. Results from the pig exposed to the only wild-type virus were partially reported in our previous study (22). All animals were housed in identical isolated rooms at VIDO. Housing conditions and diet were the same for all pigs. To compare *in utero* infection phenotypes and fetal immunopathology caused by different Zika virus variants, *in utero* inoculation was performed in pigs at 50 gestation days (gd) (the total duration of porcine pregnancy is 114–115 days) as previously described (23–26). For precise inoculation, we used an ultrasound-guided technique, which allows verifying fetal viability before and after injection by visualizing the heart beating. We inoculated four conceptuses (a fetus with fetal membranes) per pig. Animals in corresponding groups were inoculated with 2 x 10^5^ TCID_50_ per conceptus of Zika virus variants intraperitoneally + intra-amniotic (100 μl + 100 μl). The control pig was inoculated with virus-free media. Pregnant pigs were euthanized and sampled 28 days after *in utero* inoculation. Uteri with fetuses were removed. Amniotic fluid and uterine wall with the placenta (fetal placental compartment was subsequently dissected from the maternal endometrium) were collected from each conceptus and rapidly frozen. Amniotic fluid was aspirated with sterile syringes with needles before tissue dissection. All samplings were performed in the direction from non-manipulated fetuses closest to the right uterine horn tip toward inoculated fetuses. Fetuses were visually examined. Blood plasma was collected from all fetuses; after blood centrifugation (2000g, 20 min, +4°C), plasma was aliquoted and frozen (-80°C). Amniotic fluid, inguinal lymph node, thymus, spleen, and liver were sampled from all fetuses. Fetal brains were removed from all fetuses and frozen in liquid nitrogen. Body and brain weights of viable fetuses were measured. Selected fetal samples were tested by PCR for porcine circovirus 2 (PCV 2), porcine circovirus 3 (PCV 3), PRRSV and PPV (26, 27). All samples were negative for tested viruses.

### RNA extraction and reverse transcriptase quantitative polymerase chain reaction

The lateral surface of the frozen right cerebral hemisphere in adult mice and pig fetuses were shaved with sterile scalpel blades to collect 20-40 mg of tissue. Tissue samples were kept on dry ice and weighed on analytical balances. One ml of TRI Reagent Solution (Invitrogen) was added, and tissues were homogenized using RNase-free stainless-steel beads and TissueLyser II (QIAGEN) operating for 5 min at 25 Hz. Then RNA extraction was performed with PhaseMaker tubes (Invitrogen) and PureLink RNA Mini Kit (Invitrogen) according to the manufacturer’s instructions. For porcine placenta, amniotic fluids, lymph nodes, thymus, spleen, liver and mouse spleen, PureLink RNA Mini Kit was used to extract RNA according to the manufacturer’s instructions.

Zika virus-specific SYBRgreen-based one-step RT-qPCR (28) was conducted on the StepOne Plus platform (Life Technologies) and analyzed using StepOne 2.3 software. Reaction mixture (20 μl) consisted of 10 μl 2x SensiFAST SYBR Hi-ROX One-Step Mix (Bioline BIO-73005), 0.4 μl RiboSafe RNase Inhibitor, 0.2 μl reverse transcriptase, 0.8 μl (400 nM) of each primer (ZIKV-F10287: 5′-AGGATCATAGGTGATGAAGAAAAGT-3′; ZIKV-R10402: 5′-CCTGACAACACTAAGATTGGTGC-3′), 3.8 μl nuclease-free water and 4 μl RNA template. A reverse transcription step of 10 min at 45 °C and an enzyme activation step of 2 min at 95 °C were followed by 40 amplification cycles (5 s at 95 °C and 34 s at 60 °C). PCR values and a standard curve were used to quantify viral RNA loads which were corrected for RNA dilution factor in the PCR reaction, individual fluid sample volumes or individual tissue sample weights and upon logarithmical transformation expressed as Zika virus RNA genome copies per ml or gram. The standard curve was used to determine detection and quantification limits as previously described (29). The standard curve had a wide dynamic range (10^2^−10^9^ virus RNA copies/reaction for the limit of quantification) with a high linear correlation (R^2^ = 0.9997) between the cycle threshold (Cq) value and template concentration. The slope of the standard curve (-3.4351) corresponded to the 95.5% reaction efficiency level. All samples with PCR values below the limit of quantification (LOQ) had values below the limit of detection (LOD; 10 virus RNA copies/reaction); thus, only LOQ is represented in graphs. In all PCR tests, we used VERO cell culture media containing Zika virus as a positive PCR control. As a negative control, we used samples from mock-inoculated control animals. Strict precautions were taken to prevent PCR contamination. Aerosol-resistant filter pipette tips and disposable gloves were used. Kit reagent controls were included in every RNA extraction and PCR run.

### Serology

To quantify Zika virus-specific IgG antibodies in mouse and pig blood plasma, we used immunoperoxidase monolayer assay (IPMA) (23–26, 30). Briefly, VERO cells in 96-well cell culture plates were inoculated with 50 μl media containing 5 TCID_50_ of Zika virus and incubated for 2 h (+37 °C, 5% CO_2_). Then 100 μl of the culture medium (DMEM supplemented with 5% FCS, 1x Penicillin/Streptomycin, 2.67 mM Sodium Bicarbonate) was added and after incubation (72 h, +37 °C, 5% CO_2_) plates were dried and stored at -20 °C until use. Plates were thawed and cells were fixed in 10% buffered formalin (30 min, RT). Cells were washed twice with 1x DPBS (pH 7.2) and incubated with 100% methanol in the presence of 0.3% H_2_O_2_ (10 min, RT). Then plates were washed with DPBS and two-fold serial dilutions of blood plasma were added, followed by 1h incubation (+37 °C). Plates were washed three times with DPBS containing 0.05% Tween-80 and 50 μl/well of rabbit anti-pig IgG (1:400, Abcam, ab136735) or goat anti-mouse IgG (1:2000, Abcam, ab97023) antibodies conjugated with horseradish peroxidase were added. After incubation (1 h, +37 °C) and washing, color reaction was initiated by adding substrate solution (1 mM 3-amino-9-ethylcarbazole, 5% N, N-dimethylformamide, 50 mM Sodium Acetate (pH 5.0), 10 mM H_2_O_2_). The reaction was stopped by replacing the substrate with an acetate buffer and Zika virus-specific staining was determined by examination with a microscope. The titers were defined as the log reciprocal of the highest serum dilution. Blood plasma from mock-inoculated control animals was used as a negative control.

### Bio-Plex assay

Bio-Plex assay reagents are listed in **Supplementary Table 1**. We measured porcine interleukin-1*β* (IL-1*β*), interleukin-6 (IL-6), interleukin-8 (IL-8), interleukin-10 (IL-10), interleukin-12 (IL-12), interleukin-13 (IL-13), interleukin-17A (IL-17A), interferon gamma (IFN-γ), and interferon alpha (IFN-α) in blood plasma and amniotic fluid as previously described (26, 31). Briefly, Bio-Plex bead coupling was performed as per the manufacturer’s instructions. The multiplex assay was carried out in a 96 well Grenier Bio-One Fluotrac 200 96F black plates (VWR). The beadsets conjugated with the capture antibodies were vortexed for 30 s followed by sonication for another 30 s to ensure total bead dispersal. The bead density was 1200 beads/μl in PBS-BN (1 × PBS pH 7.4 + 1% bovine serum albumin (Sigma-Aldrich) + 0.05% sodium azide (Sigma-Aldrich). One μl of each beadset was added to diluent (PBS + 1% porcine serum + 0.05% sodium azide) for a total volume of 50 μl per well. The plate was then washed using the Bio-Plex Pro II Wash Station (BioRad; washed twice with 100 μl PBST (phosphate buffered saline with Tween 20). The protein standards with starting concentrations as indicated in the table were mixed in a diluent and 2.5-fold dilutions were tested to produce the standard curve. Fifty μl per well of each dilution was added to the plate. Blood plasma samples were prediluted 1:4, amniotic fluid diluted 1:2 and added to the wells at fifty μl per well in duplicate. The plate was agitated at 800 rpm for 1 h at room temperature. After 1 h incubation with the samples, the plate was washed (3 × 150 μl PBST). Fifty μl of a biotin cocktail (consisting of the biotins diluted as indicated in **Supplementary Table 1**) was added to each well. The plate was resealed, covered and agitated at 800 rpm for 30 min at room temperature then washed again as indicated above. Fifty μl of Streptavidin RPE (Prozyme; diluted to 5 μg/ml) was added to each well. Then the plate was resealed, covered and agitated at 800 rpm for 30 min at room temperature and washed as indicated above. A hundred μl of 1 × Tris-EDTA was added to each well and then the plate was vortexed for 5 min before reading on the BioRad Bio-Plex 200 instrument following the manufacturer’s instructions. The instrument was set up to read a minimum of 60 beads in appropriate regions for each beadset (**Supplementary Table 1**). Results were corrected by subtracting the background levels, expressed as median fluorescent intensity, and analyzed using Bio-Plex Manager software with 5PL curve fitting.

### Histopathology

Mouse brain tissues were dissected and fixed in formalin for subsequent hematoxylin and eosin (H & E) staining to screen lesions.

### Next-generation sequencing

RNA from virus stocks, brain, and placenta samples were used to construct Zika virus whole-genome NGS libraries; libraries for mouse brain and porcine placental samples were constructed independently. While we found Zika RNA in lymph nodes from 84.6% of porcine fetuses in the wild-type group and 42.8% of fetuses in the E/NS1+176CpG group, viral loads in E/NS1+176CpG fetuses were not sufficient to construct NGS libraries. Thus, we only used mouse brain and porcine placental samples for evolutionary analysis. We used the PrimalSeq protocol, an approach wherein a nearly whole virus genome is amplified in the ~400–480 bp overlapping fragments with multiplexed PCR reactions and with two technical replicates for each multiplex reaction (32). The amplicons from multiplex PCR reactions are combined for library preparation and NGS. Zika virus RNA was reverse transcribed into cDNA using ProtoScript^®^ II Reverse Transcriptase (20 μL reactions; New England Biolabs) and Random Hexamers (2 μL; Thermo Fisher Scientific). As previously validated for accurate SNV calling, we used more than 1,000 viral RNA copies for cDNA synthesis (32). Zika virus cDNA (2 μL) was amplified in two multiplexed Zika-specific PCR reactions with Primal Scheme primers (**Supplementary Table 2**). These two multiplex primer schemes for wild-type Zika virus variant were previously designed with a web-based tool, Primal Scheme, for the Zika virus PRVABC59 strain (GenBank: KU501215.10) (32). To account for genomic differences between the Zika virus PRVABC59 strain and the H/PF/2013 strain used in this study, we modified ZIKA_400_1_LEFT, ZIKA_400_17_RIGHT and ZIKA_400_32_LEFT primers, and added the ZIKA_400_36_LEFT and ZIKA_400_36_RIGHT primer pair (**Supplementary Table 2**). To account for *de novo* introduced CpG dinucleotides in Zika virus variants, we designed a specific set of primers (**Supplementary Table 2**). Altogether, the primer pairs amplified 350–480 nt products with a nearly 100 nt overlap covering almost the entire Zika virus genomes. Each multiplexed reaction was performed in two technical replicates with Q5^®^ Hot Start High-Fidelity 2× Master Mix (25 μL reactions; New England Biolabs). An enzyme activation step of 30 s at +95°C was followed by 35 amplification cycles for 15 s at +95°C and 5 min at +65°C. The correct size of amplicons in each reaction was verified by agarose gel electrophoresis.

We constructed libraries and made Illumina sequencing as previously described with some modifications (32). Virus amplicons from the two multiplex PCR reactions were combined (separately for each technical cDNA replicate) and purified using magnetic beads (1.8:1 ratio of beads (45 μL) to combined sample (25 μL)). DNA concentrations were measured with the Qubit dsDNA HR Assay Kit and Qubit 3.0 Fluorometer (Thermo Fisher Scientific). In total, 50 ng of purified DNA in 60 μL was used for library construction with the NEBNext^®^ Ultra™ II DNA Library Prep Kit for Illumina^®^ (NEB) and NEBNext^®^ Multiplex Oligos for Illumina^®^ (96 Index Primers) (NEB) according to the manufacturer’s protocol. Individual sample libraries were quantified with the Qubit dsDNA HR Assay Kit and Qubit 3.0 Fluorometer. The expected peak DNA fragment size (~580 bp) was confirmed with the Agilent DNA 1000 kit on Agilent 2100 Bioanalyzer (Agilent). In total, 20 ng of each barcoded library was pooled, quantified (Qubit 3.0), quality-checked (Agilent Bioanalyzer), and converted to moles: Molecular weight [nM] = Library concentration [ng/µL]/((Average library size x 650)/1,000,000). The pooled library was diluted to 2 nM in 10 mM TE, denatured with 0.1 N NaOH and diluted to 14 pM, and paired-end 300 nt reads were generated with MiSeq Reagent kit v3 600 (Illumina) on the MiSeq System (Illumina). Access to sequencing data files generated in this study is available in the Sequence Read Archive (SRA) database under BioProject ID PRJNA837685.

### Illumina data processing and variant calling with iVar

We used an open-source software package iVar (Intra-host Variant Analysis from Replicates) to process virus sequencing data and call single nucleotide variations (SNVs) (32). To identify SNVs, we compared Zika virus sequences from experimental samples to a reference sequence. As references for Zika virus variant stock sequences and sequences from *in vivo* samples, we used ancestral wild-type, E+102CpG, and E/NS1+176CpG sequences which were used to synthetically generate Zika virus variants (10) (**Supplementary File 1**). Synonymous and non-synonymous mutations not present in the reference sequence were considered SNVs. Then, we compared the genomic positions and frequencies of SNVs between sequences from the initial Zika virus stocks used for animal inoculation and sequences from *in vivo* samples; tissue-specific SNVs that were not present in initial corresponding Zika virus stocks were considered as *de novo* emerged intra-host SNVs (iSNVs).

As per the previously described and validated best practices (32), we analyzed virus genomic regions with a sequencing depth of at least 400×. Only SNVs/iSNVs with a frequency of at least 3% detected in both technical replicates were considered for analysis; insertions and deletions were not analyzed. In addition, the genomic regions amplified with primers that contained nucleotide mismatches within the binding sites were omitted. The detailed computational protocol used in this paper for iVar was previously published (22).

### Statistical analysis

We used GraphPad PRISM 8 software. Results with a statistically significant difference had p < 0.05. All data were expressed as individual values, mean, or median. The groups inoculated with different Zika virus variants were compared with the negative control group and between each other. In the mouse study, the Zika virus loads in tissues and antibody titers were compared with two-way ANOVA and Dunnett’s multiple comparisons test as well as with the Kruskal-Wallis *H*-test and Dunn’s multiplicity-adjusted post-test. The bodyweight dynamics were compared using a mixed-effects model. In the porcine model study, the number of dead fetuses was compared with the Yates-corrected χ^2^-test. Brain to body weight ratio, Zika virus loads in tissues and cytokine levels in the porcine fetuses were compared with the Kruskal-Wallis *H*-test and Dunn’s multiplicity-adjusted post-test. The correlation of IFN-a and IFN-γ levels in fetal body fluids with Zika virus loads in amniotic fluids was assessed with the Spearman correlation test. Genomic complexity between sequences from mouse brains and porcine fetuses inoculated with different Zika virus variants was compared with the Kruskal-Wallis *H*-test.

## Results

### Infection in the mouse brain

**Figure 1:**
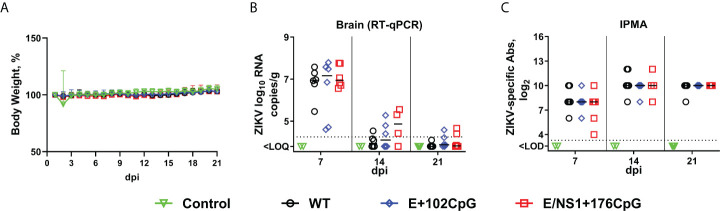
Zika virus infection kinetics in mice. **(A)** Body weight. **(B)** Zika virus loads in the brain. **(C)** Zika virus-specific IgG antibodies quantified by immunoperoxidase monolayer assay (IPMA). Dotted lines represent the limit of quantification (LOQ) or detection (LOD). The LOQ was established by calculating the lowest possible virus load determined by the linear standard curve, adding RNA dilution factor in the PCR reaction, and the log_10_-transformed dilution factor of the tissue sample with the lowest weight. The LOQ for brain samples—10^4.3^ RNA copies/g. Solid lines represent median values. dpi: days post inoculation. WT: wild-type Zika virus. In the mouse study, the Zika virus loads in tissues and antibody titers were compared with two-way ANOVA and Kruskal-Wallis *H*-test tests. The bodyweight dynamics were compared using a mixed-effects model.

To model the scenario where recoded vaccine strains breach the blood-brain barrier and compare infection phenotypes caused by wild-type and CpG-recoded Zika virus variants in the brain, we injected adult mice intracerebrally with 10^3^ TCID_50_ of wild-type, E+102CpG, or E/NS1+176CpG Zika virus variants. Mice did not show changes in behavior and appearance. The body weight did not differ significantly between groups (**Figure 1A**; p = 0.83). At 7 days after inoculation, Zika virus loads in brain tissues were similarly high and values ranged from 4.60 to 7.79 log_10_ RNA copies/g (**Figure 1B**). At 14 and 21 days after inoculation, Zika virus loads lowered in all groups (**Figure 1B**). We did not detect virus in blood plasma and spleen. Mice in all groups showed high virus-specific antibody responses (**Figure 1C**). There was no statistically significant difference in viral loads and antibody titers between groups (p = 0.19 and p = 0.73; **Figures 1B**, **C**), or between female and male mice (p = 0.70 and p = 0.43).

**Figure 2:**
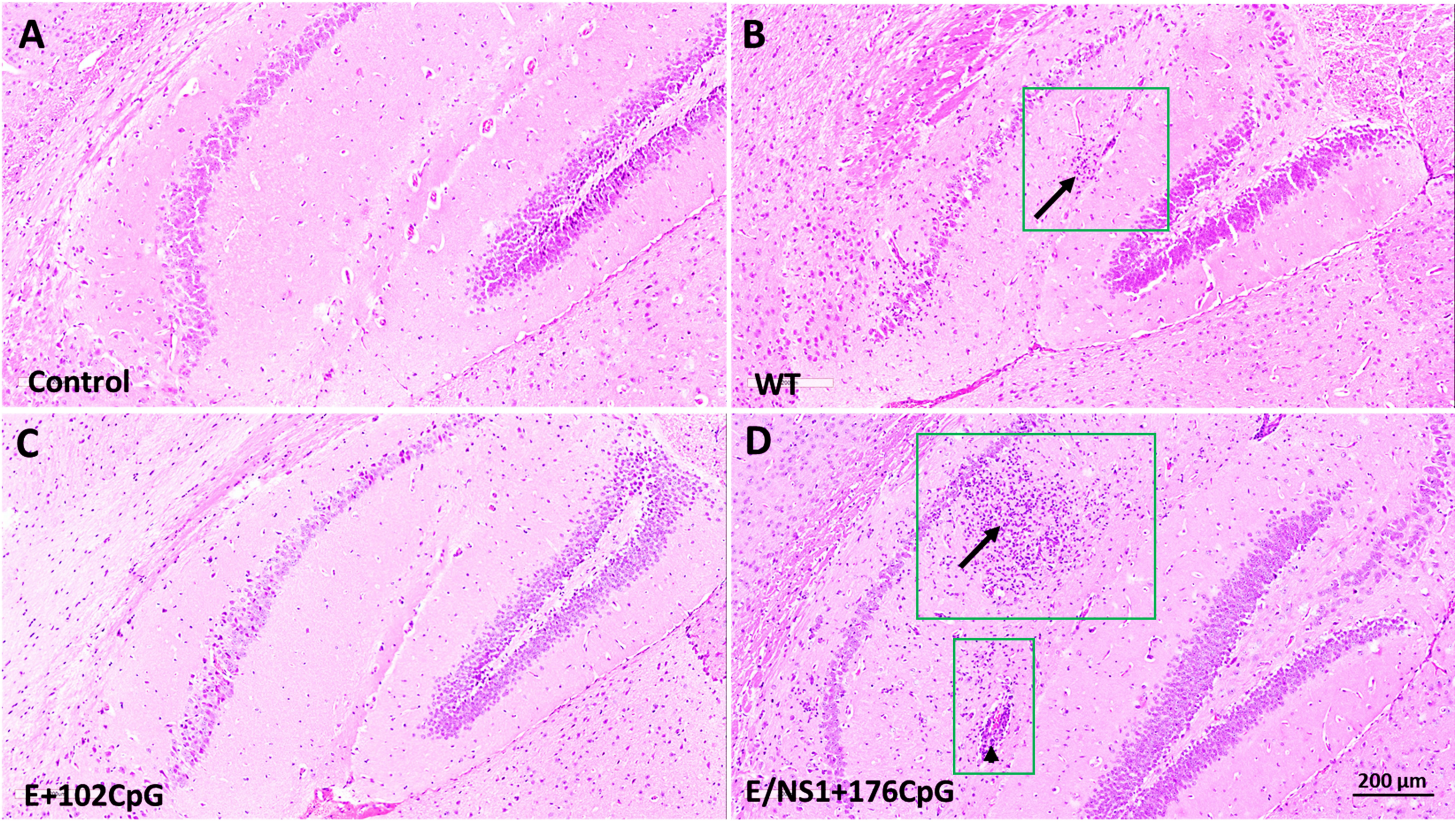
Histopathology in brains of mice infected with different Zika virus variants. Representative images of H & E staining in brain sections. **(A)** Mock inoculated control mice with no lesions. **(B)** Wild-type (WT) infected mice; lesions were detected at 14 and 21 days after inoculation in 50% of animals. Hippocampal cell invasion (arrow within a green square). Inflamed blood vessel (arrowhead within a green square). **(C)** E+102CpG infected mice with no lesions. **(D)** E/NS1+176CpG infected mice; lesions were detected at 21 days after inoculation in 40% of animals. Hippocampal cell invasion (arrow within a green square). Brains from control and experimental groups (n = 5-7 mice per group) were tested; two sections were analyzed from each sample.

While animals did not show a significant difference in viral loads, histological lesions differed. Mice in wild-type and E/NS1+176CpG groups had brain histopathology represented by cell infiltration in the hippocampus and blood vessel inflammation (**Figures 2B**, **D**) consistent with previously described histopathology in Zika virus-infected mouse brains (10, 33, 34). Conversely, the mock-inoculated Control and E+102CpG mice did not have histopathology in the brain (**Figures 2A**, **C**).

To summarize, all Zika virus variants had similar infection kinetics in mouse brains. However, in contrast to the wild-type and E/NS1+176CpG variants, the recoded E+102CpG variant showed the different phenotype with no lesions in the brain.

### Evolution of wild-type and CpG-recoded Zika virus variants in the brain

Comparable RNA loads caused by wild-type and CpG-recoded Zika virus variants in mouse brains (**Figure 1B**) provided an opportunity to compare the evolution of variants *in vivo*, which is challenging to accomplish in conventional models where after peripheral inoculation recoded variants show attenuated phenotypes with low or no viral loads (10).

The initial Zika virus stock and brain samples had specific DNA bands after amplification in multiplex PCR, 77.8-96.4% of virus genome coverage, and a high (3,739-26,658×) average sequencing depth (**Supplementary Tables 3, 4**; **Supplementary File 2**).

Comparing sequences from virus stocks and brain samples with the corresponding parental reference sequences (**Supplementary File 1**; these sequences were used to *de novo* synthetize DNA fragments to rescuer variants) let us identify Zika virus stock- and variant-specific SNVs (**Supplementary Table 5**). We used iVar for NGS processing and variant calling with a sequence depth of at least 400×, a frequency of at least 3%, and only variants detected in both technical replicates (32). Mutations in the E+102CpG variant (C1675T; brain sample IC-13L) and in the E/NS1+176CpG variant (C2280A; brain sample IC-4-2R) changed one out of 102 and 176 *de novo* introduced CpG dinucleotides in each recoded variant. All other *de novo* introduced CpG dinucleotides were preserved in the E+102CpG and NS1/E+176CpG sequences showing substantial stability of recoded variants during persistent infection in the brain (**Supplementary Table 5**).

**Figure 3:**
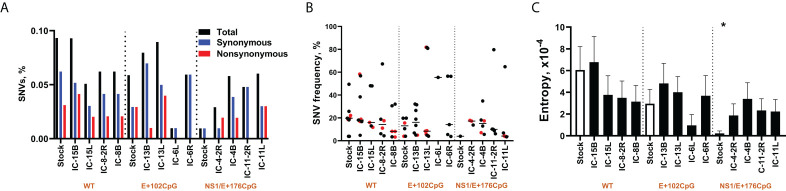
Zika virus evolution in the mouse brain. **(A)** The mean percentage (from two technical replicates) of Zika virus genomic single nucleotide variations (SNVs) in virus stock and mouse samples was calculated from the total number of sequenced nucleotides in each sample. **(B)** The mean frequency (from two technical replicates) of Zika virus genomic SNVs in virus stock and mouse samples. Black circles—synonymous SNVs. Red circles—non-synonymous SNVs. Solid lines represent median values. **(C)** To estimate complexity, Shannon’s entropy was calculated for each site to reflect Zika virus genetic complexity as previously described (32). A statistically significant difference (p = 0.02; Kruskal-Wallis test) was found for the E/NS1+176CpG variant stock compared to the wild-type (WT) Zika virus stock. Raw NGS data are shown in **Supplementary Tables 3**–**5**.

The initial virus stock of wild-type Zika virus contained 0.09% of SNVs, as calculated from the total number of sequenced nucleotides (**Figure 3A**). The E+102CpG and E/NS1+176CpG Zika virus stocks had fewer SNVs: 0.06% and 0.01%. The percentage of SNVs was somewhat preserved in brain samples in the wild-type (0.05-0.09%) and E+102CpG groups (0.01-0.09%), and increased to comparable levels in the NS1/E+176CpG group (0.03-0.06%) (**Figure 3A**).

All stock-specific mutations were preserved during infection and evolution in mouse brains in the wild-type Zika virus group (**Supplementary Table 5**). Most stock-specific mutations were conserved during infection and evolution in mouse brains in the E+102CpG Zika virus group except two G9196T and C9198G mutations (**Supplementary Table 5**). The single stock-specific mutation in E/NS1+176CpG Zika virus disappeared during infection in mouse brains. The numbers of SNVs and ratios of synonymous/non-synonymous mutations in the corresponding virus stock and brain samples were similar for the wild-type and E+102CpG Zika virus groups (**Figure 3B**). In contrast, mice from the E/NS1+176CpG Zika virus group had an increased number of SNVs (from 1 SNV in the stock to 3-6 SNVs in brain samples) and had a 38 ± 28% ratio of synonymous/non-synonymous mutations in sequences from brain samples.

The genomic complexity was significantly lower in the initial E/NS1+176CpG Zika stock than in stocks of other variants (p = 0.02, Kruskal-Wallis test; **Figure 3C**). However, genomic complexity was not statistically different between sequences from mouse brains inoculated with different Zika virus variants (p = 0.78-0.99, Kruskal-Wallis test).

Next, to identify *de novo* emerged intra-host brain-specific mutations, we compared Zika virus sequences from brain samples with the NGS sequences from corresponding virus stocks. Only mutations in brains that were not present in the initial virus stock were considered as *de novo* emerged iSNVs. We found 3-4, 5, and 3-6 iSNVs per mice in wild-type, E+102CpG, and E/NS1+176CpG groups (**Supplementary Table 5**). All iSNVs were unique, and most of the iSNVs were positioned in non-structural proteins (**Supplementary Table 5**). To further confirm that iSNVs are not virus stock-derived artifacts, we called variants in the Zika virus stock sequences with the low-frequency threshold (0.01%) and compared low-frequency stock-specific SNVs to brain-specific iSNVs. This threshold is much below the sequencing error threshold reported for Illumina equipment—0.1-0.46% (35–37). All iSNVs in Zika stock sequences were much below the 0.1-0.46% error threshold (**Supplementary Table 5**). Only one brain-specific iSNV in a mouse from the NS1/E+176CpG group had a frequency above the threshold—1.21% (A1344G). Thus, most identified iSNVs emerged *de novo* in mouse brains and one was selected and amplified from low-frequency stock-specific mutation. Finally, to confirm that virus genome coverage did not affect analysis—i.e., when brain-specific iSNVs are virus stock-derived artifacts, but coverage gaps in Zika virus stock sequences bias analysis—we compared NGS coverage gaps in sequences from virus stocks and *in vivo* samples (**Supplementary Table 4**). Most coverage gaps were identical between sequences from the virus stock and *in vivo* samples, and gaps did not affect iSNV analysis (**Supplementary Table 4**).

Altogether, most *de novo* introduced CpG dinucleotides were preserved in sequences of recoded Zika virus variants during infection in mouse brains showing the stability of vaccine viruses in the present study. However, to better understand the stability of *de novo* introduced CpG dinucleotides in virus genomes, *in vivo* studies with serial passaging are needed. While wild-type and CpG-recoded virus stocks that were used for mouse inoculation initially showed differences in the number of SNVs, *in vivo* evolution resulted in comparable mutation frequencies and genomic entropy.

### *In utero* infection

To model the scenario where recoded vaccine strains breach the placental barrier and to compare *in utero* infection phenotypes caused by wild-type and CpG-recoded Zika virus variants, we used the well-established fetal pig model which reproduces key aspects of persistent *in utero* Zika virus infection in humans (23–26). Importantly, pigs have the same level of genomic CpG dinucleotide suppression similar to humans and mice. Pigs, on average, have 12-16 fetuses; inoculation of four fetuses closest to the tip of the uterine horn results in trans-fetal virus spread and persistent infection in directly inoculated, adjacent, and distant siblings that allows to compare *in utero* infection kinetics of different Zika virus variants (24–26).

**Figure 4:**
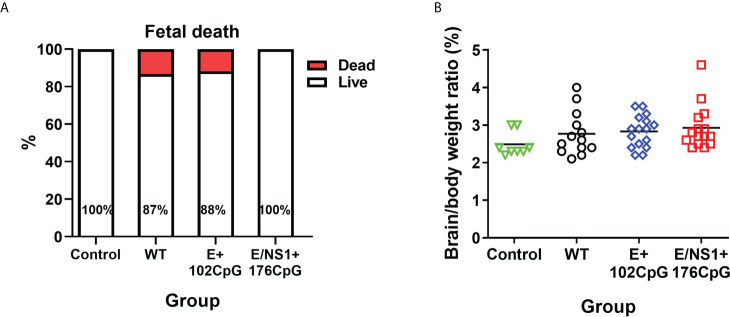
Fetal death and brain/body ratio. **(A)** Percentage of live and dead fetuses. **(B)** Fetal brain/body weight ratio in control and Zika virus-exposed litters. Solid lines represent mean values. WT: wild-type Zika virus. The number of dead fetuses was compared with the Yates-corrected χ^2^-test. Brain to body weight ratio were compared with the Kruskal-Wallis *H*-test and Dunn’s multiplicity-adjusted post-test.

Zika virus variants caused persistent silent *in utero* infection without overt fetal pathology and death, which allowed comparison of *in utero* infection kinetics. Litters infected with wild-type and E+102CpG variants had 12-13% mummified fetuses (**Figure 4A**) which is in line with reported typical fetal death rates in pig pregnancy (38, 39); having the typical fetal death rates, we cannot conclude whether Zika infection contributed to the fetal death in this study. Control and E/NS1+176CpG litters were free of dead fetuses. The brain/body weight ratios were similar across all groups (**Figure 4B**).

We did not detect Zika variants in the fetal thymus, spleen, and liver tissues. Brains in all fetuses in all groups were also negative for the virus. In all our previous studies with the patient-derived wild Asian PRVABC59 strain [GenBank: KU501215.1], Zika virus caused infection in fetal brains (24, 26). In this study, synthetically-derived wild-type Asian H/PF/2013 Zika virus [GenBank: KJ776791.2] and CpG-recoded variants did not cause infection in fetal brains. It is possible that H/PF/2013 strain and H/PF/2013-derived CpG variants did not cross the fetal blood-brain barrier or replicated at lower levels and for a shorter time in the fetal brain. Accordingly, H/PF/2013 strain caused less disseminated *in utero* infection than the PRVABC59 strain in baboons (40). In addition, we used synthetically-derived H/PF/2013 Zika virus variants; potentially, different mutant spectrum complexity between patient-derived (with higher complexity in the initial inoculum) and *de novo* variants (with lower complexity in the initial inoculum) could also contribute to the discrepancy. In support, in our ongoing unpublished study, synthetically-derived wild-type PRVABC59 variants, which have lower complexity than the patient-derived parental PRVABC59 strain, also do not cause fetal brain infection. In contrast, the patient-derived parental PRVABC59 strain causes infection in fetal brains.

**Figure 5:**
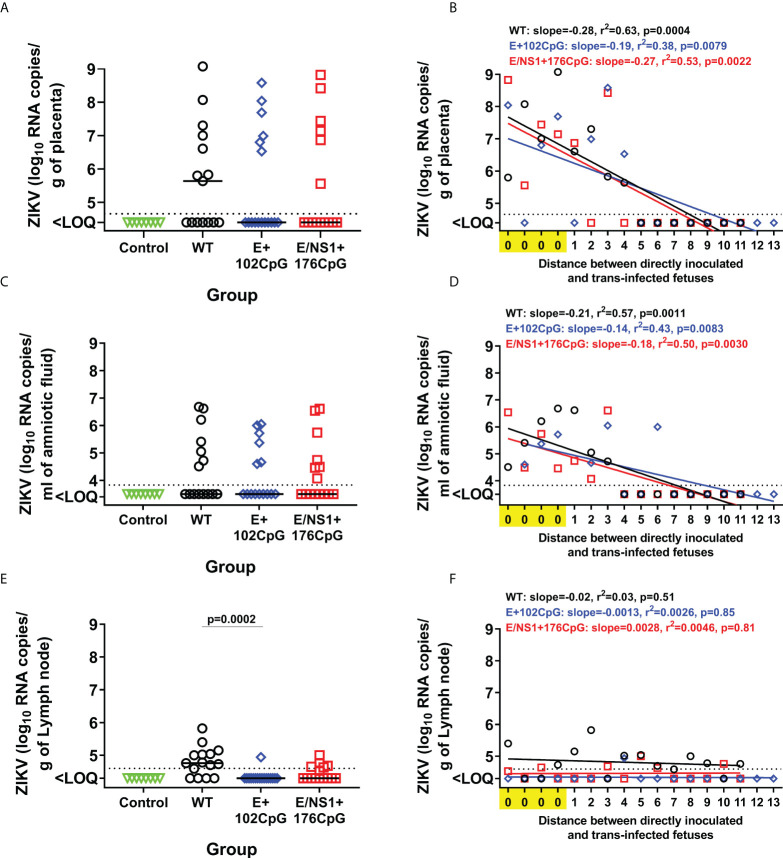
*In utero* infection phenotypes of wild-type and CpG-recoded Zika virus variants in the fetal pig transmission model. Zika virus RNA loads in the placenta **(A)**, amniotic fluid **(C)**, and lymph nodes **(E)** of directly inoculated and not manipulated trans-infected fetuses. Solid lines represent median values. Linear regression shows the relationship between Zika virus loads in placenta **(B)**, amniotic fluid **(D)**, and lymph nodes **(F)**, and the distance between directly inoculated and trans-infected siblings in the uterus. The x-axis for linear regression modeling: 0 – a directly inoculated index fetus; 1-13 is the distance from the directly virus-inoculated fetuses to a non-manipulated sibling. Lines represent the fitted regression. The dotted line represents the limit of quantification (LOQ). The LOQ was established by calculating the lowest possible virus load determined by the linear standard curve, adding RNA dilution factor in the PCR reaction, and the log_10_-transformed dilution factor of the tissue sample with the lowest weight. The LOQ for placenta—10^4.6^ RNA copies/g; LOQ for amniotic fluids—10^3.8^ RNA copies/ml; LOQ for lymph node—10^4.6^ RNA copies/g. ZIKV: Zika virus. WT: wild-type. Zika virus loads in tissues were compared with the Kruskal-Wallis *H*-test and Dunn’s multiplicity-adjusted post-test.

All Zika virus variants caused similar loads in the placenta and amniotic fluid (**Figures 5A**, **C**). *In utero* infection kinetics were also similar: Regression analysis of variant loads in the placenta and amniotic fluid from directly inoculated and trans-infected fetuses showed similar efficiency of virus transmission between siblings within the uterus (**Figures 5B**, **D**). In contrast, the difference in virus loads and infection kinetics was apparent in fetal lymph nodes. Wild-type Zika virus caused high loads in lymph nodes collected from 84.6% of fetuses (**Figures 5E**, **F**). The E/NS1+176CpG recoded variant caused infection in lymph nodes from 42.8% of fetuses (**Figures 5E**, **F**). And the E+102CpG variant was detected in only one fetus (6.3%; **Figures 5E**, **F**).

**Figure 6:**
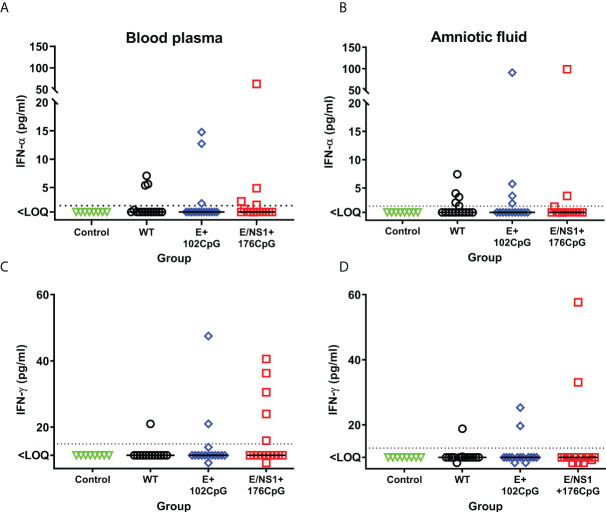
Fetal IFN-α and IFN-γ responses. IFN-α levels in fetal blood **(A)** and amniotic fluid **(B)**. IFN-γ levels in fetal blood **(C)** and amniotic fluid **(D)**. Solid horizontal lines represent median values. The dotted line represents the limit of quantification (LOQ). WT: wild-type Zika virus. Cytokine levels in the porcine fetuses were compared with the Kruskal-Wallis *H*-test and Dunn’s multiplicity-adjusted post-test.

In addition to *in utero* infection kinetics, we compared cytokine responses in fetuses. Type I interferon *in utero* responses to virus infection were suggested as a major pathological factor affecting fetal and offspring health (24, 26, 41–43). Thus, we compared *in utero* IFN-α responses between litters exposed to Zika virus variants and Control fetuses. The wild-type and all recoded variants induced increased IFN-α levels in fetal blood when compared with mock-inoculated control fetuses (**Figure 6A**). Similar trends were also observed in the amniotic fluids (**Figure 6B**). The levels of IFN-α in fetal blood (wild-type: r = 0.53, p = 0.057; E+102CpG: r = 0.66, p = 0.011; E/NS1+176CpG: r = 0.76, p = 0.0018) and amniotic fluid (wild-type: r = 0.77, p = 0.0015; E+102CpG: r = 0.62, p = 0.012; E/NS1+176CpG: p = 0.38) correlated with Zika virus loads in amniotic fluids.

Interestingly, some fetuses exposed to recoded Zika virus E+102CpG and particularly E/NS1+176CpG variant showed a trend to higher IFN-γ levels—cytokine critical for immunity against virus infections and eliciting vaccine responses—in comparison to fetuses exposed to the wild-type variant (**Figures 6C**, **D**); however, a statistically significant difference was not observed (amniotic fluid: p > 0.99; blood plasma: p > 0.99). In contrast to IFN-α, the levels of IFN-γ in fetal blood (wild-type: r = -0.28, p = 0.69; E+102CpG: r = 0.00, p > 0.99; E/NS1+176CpG: r = 0.30, p = 0.28) and amniotic fluid (wild-type: r = -0.20, p = 0.46; E+102CpG: r = 0.24, p = 0.35; E/NS1+176CpG: r = -0.20, p = 0.47) did not correlate with Zika virus loads in amniotic fluid.

**Figure 7:**
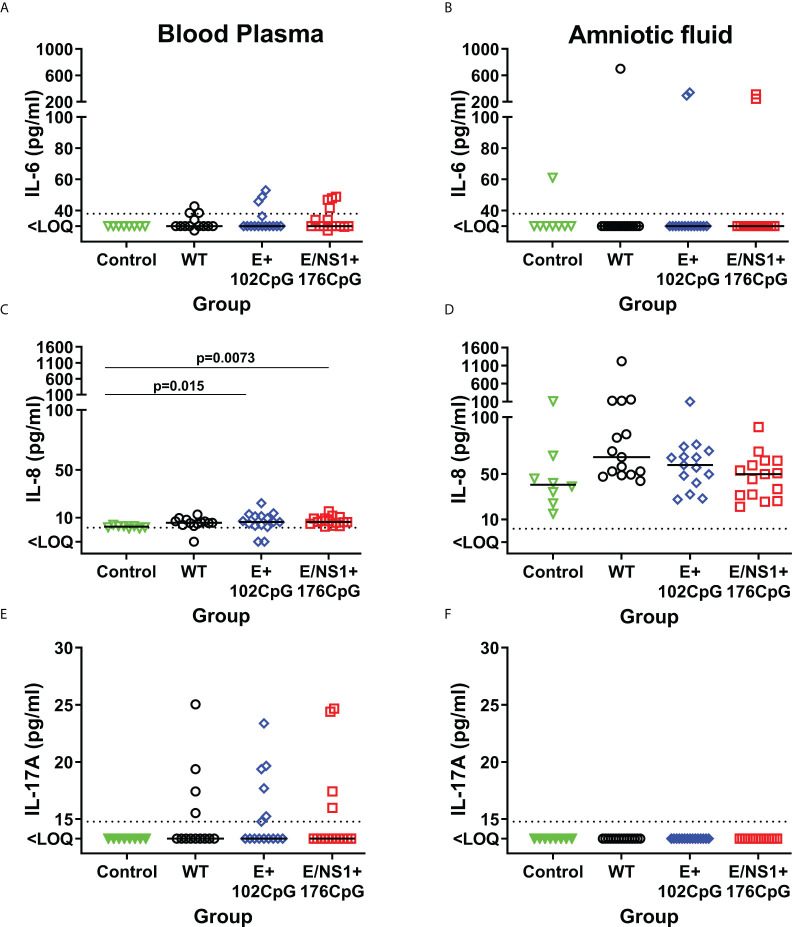
Cytokines in fetal blood plasma and amniotic fluids. Solid horizontal lines represent median values. The dotted line represents the limit of quantification (LOQ). WT: wild-type Zika virus. Cytokine levels in the porcine fetuses were compared with the Kruskal-Wallis *H*-test and Dunn’s multiplicity-adjusted post-test.

In addition to IFN-α and IFN-γ, we also compared IL-1*β*, IL-6, IL-8, IL-10, IL-12, IL-13, and IL-17A levels. Levels of IL-1*β*, IL-12, IL-10 and IL-13 were below the detection limits in both blood plasma and amniotic fluids collected from Control and Zika fetuses. Some fetuses infected with Zika virus variants had increased IL-6, IL-8, and IL-17A in blood plasma and amniotic fluids (**Figure 7**), but with no statistically significant difference.

To summarize, Zika virus variants had different infection kinetics in fetal lymph nodes. Also, fetuses exposed to recoded variants showed a trend to higher IFN-γ responses than fetuses exposed to wild-type Zika virus.

### Evolution of wild-type and CpG-recoded Zika virus variants *in utero*

The initial Zika stock and placental samples had correct DNA bands after amplification in multiplex PCR, 98.2-96.4% of virus genome coverage, and a high (6,687-22,597×) average sequencing depth (**Supplementary Tables 3, 4**; **Supplementary File 3**).

We compared sequences from virus stock and placental samples with the corresponding parental reference sequences (**Supplementary File 1**; only samples from wild-type and E/NS1+176CpG were studied in the *in utero* experiment because E+102CpG and E/NS1+176CpG variants persisted with the same viral loads in the placenta suggesting similar evolutionary pressure, **Figures 5A**, **B**). Two mutations in the E/NS1+176CpG variant (G1825A and C2324T; in placental samples 313-8-F14 and 313-8-F13) affected two out 176 *de novo* introduced CpG dinucleotides. All other *de novo* introduced CpG dinucleotides were preserved showing the substantial stability of the recoded variant during persistent infection in the placenta (**Supplementary Table 5**).

None of the stock-specific mutations was preserved during infection and evolution in placental tissues in the wild-type Zika virus group (**Supplementary Table 5**).

**Figure 8:**
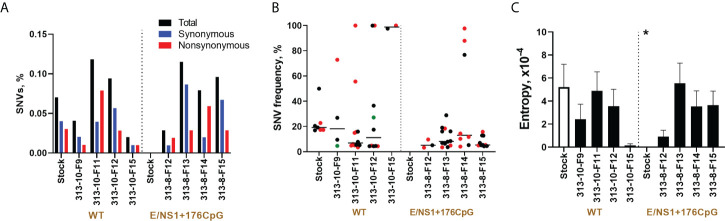
Zika virus evolution in the porcine placenta. **(A)** The mean percentage (from two technical replicates) of Zika virus genomic single nucleotide variations (SNVs) in virus stock and porcine samples was calculated from the total number of sequenced nucleotides in each sample. **(B)** The mean frequency (from two technical replicates) of Zika virus genomic SNVs in virus stock and porcine samples. Black circles—synonymous SNVs. Red circles—non-synonymous SNVs. Green circles—SNVs in untranslated regions. Solid lines represent median values. **(C)** To estimate complexity, Shannon’s entropy was calculated for each site to reflect Zika virus genetic complexity as previously described (32). A n asterisk (*) indicates a statistically significant difference (p = 0.007; Kruskal-Wallis test) was for the E/NS1+176CpG variant stock compared to the wild-type (WT) Zika virus stock. Raw NGS data are shown in **Supplementary Tables 3**–**5**.

Zika virus SNVs were identified in the initial wild-type virus stock (0.07% of SNVs, as calculated from the total number of sequenced nucleotides, **Figure 8A**), while E/NS1+176CpG stock did not have mutations (**Supplementary Table 5**). The percentage of wild-type Zika virus SNVs in placental samples varied from 0.02 to 0.12% (**Figure 8A**). The frequency of SNVs in the wild-type virus stock and placental samples varied (**Figure 8B**); the most notable difference was that in each placental sample at least one non-synonymous mutation reached 79-100% frequency (**Figure 8B**). While E/NS1+176CpG Zika virus stock had no SNVs, in placental samples the percentage of SNVs increased to the rate comparable to the wild-type variant—0.03-0.12% (**Figure 8A**). The frequency of SNVs in E/NS1+176CpG infected placental samples were mostly grouped below 20%, which is notable because the initial virus stock did not have mutations. Only three SNVs in one placental sample reached 79-100% frequency (**Figure 8B**). The ratio of synonymous/non-synonymous mutations was similar for wild-type (43% of non/synonymous mutations for stock and 43 ± 19% for placental sample) and E/NS1+176CpG (49 ± 25% for placental samples; **Figure 8B**) variants.

The genomic complexity was significantly lower in the initial E/NS1+176CpG ZIKV stock than in the wild-type stock (p = 0.007, **Figure 8C**). However, no statistically significant difference was found for virus sequences from placental samples infected with different variants (p = 0.96, Mann-Whitney test).

To identify *de novo* emerged intra-host placenta-specific mutations, we considered only SNVs that were not present in stock Zika sequences at an error threshold reported for Illumina equipment (0.1-0.46%). Placental samples infected with the wild-type and E/NS1+176CpG variants did not show stock-specific sequences (**Supplementary Table 5**), except for two mutations (wild-type A4778G—0.12%; E/NS1+176CpG C1372T—0.14%) which were at the lower Illumina error threshold. Thus, most identified iSNVs emerged *de novo* in placental tissues. The virus genome coverage did not affect the results of this analysis because coverage gaps in sequences from virus stocks and *in vivo* samples were mostly identical (**Supplementary Table 4**).

Altogether, most of the *de novo* introduced CpG dinucleotides were preserved in sequences of recoded Zika virus variants during infection in placental tissues showing the stability of the vaccine virus. While wild-type and CpG-recoded virus stocks that were used for pig inoculation initially showed differences in the number of SNVs, *in vivo* evolution resulted in comparable mutation frequencies and genomic entropy.

## Discussion

Codon deoptimized (CD) and codon pair deoptimized (CPD) viruses have been studied in mice (44, 45). All CD and CPD viruses besides containing deoptimized codons or codon pairs have increased UpA dinucleotides (that also attenuate virus infection phenotypes but to a lesser extent than CpG increase (46)), and the increased number of CpG dinucleotides (46). Thus, all *in vitro* and *in vivo* studies with CD and CPD viruses represent triple recoding effects: CD or CPD, UpA enrichment, and CpG enrichment, and cannot discriminate effects of individual recoding approaches. This triple recoding may impair an understanding of attenuating mechanisms and the development of rational recoding approaches for live vaccines. The number of *in vivo* studies with CpG-recoded viruses that are not affected by UpA recoding and codon/codon pair bias deoptimization remains limited. Here, we expanded *in vivo* infection studies with CpG-recoded viruses in two animal models—the local brain infection model after direct intracerebral injection in adult mice, and the porcine *in utero* infection model. These models may provide a better understanding of the potential safety risks of CpG-recoded live vaccines against neurotropic and congenital viruses.

The traditional rationale of CpG dinucleotide suppression in vertebrates by the methylation-deamination-mutation hypothesis cannot explain why RNA viruses also have an underrepresentation of genomic CpG dinucleotides (47). Although methylation occurs in RNA viruses, it does not lead to the depletion of CpG dinucleotides (48, 49). Mechanistically, ZAP targets recoded HIV-1 and echovirus-7 by directly binding to CpG-enriched genomic regions targeting viral RNA to degradation (8, 11). With analysis of archived gene expression data, it has been suggested that central nervous tissues express low levels of ZAP that may impair CpG-depended attenuation of virus infection (11). Here, we experimentally addressed whether the CpG-recoded neurotropic Zika virus that showed restricted replication after peripheral injection in neonatal and pregnant mice (10) may cause infection comparable to wild-type virus after direct intracerebral injection. We directly injected mice intracerebrally to compare infection phenotypes caused by wild-type and two CpG-recoded Zika virus variants and to model the scenario where a vaccine strain breaches the blood-brain barrier. Supporting the above theoretical statement (11), direct intracerebral injection with wild-type or CpG-recoded Zika virus variants led to brain infection with comparable virus loads (**Figure 1B**). In contrast, our previous study showed that infection caused by the same CpG-recoded Zika virus variants in adults was efficiently attenuated by the host after subcutaneous virus inoculation (10). We previously showed that ZAP protein expression in the mouse brain is readily detectable with specific antibodies (10). Our data suggest that ZAP co-factors (8, 10–15) required to suppress CpG-recoded viruses maybe not be expressed or are under-expressed in the brain. Future studies on how CpG-recoded viruses interact with ZAP and co-factors in cells of the nervous system are needed to better understand the safety risks of recoded vaccines against neurotropic viruses. Although it should be noted that while E+102CpG and NS1/E+176CpG variants cause brain infection in mice after direct intracerebral injection, yellow fever 17D live attenuated vaccine, one of the most successful live attenuated vaccines, causes brain infection and high mortality in immunocompetent mice (50–53). In addition, a live attenuated Japanese encephalitis virus vaccine that is successfully used to control infection in the amplifying porcine host also causes neurovirulence in mice after intracranial injection (54).

Counterintuitively, the Zika virus E+102CpG variant—the variant with the lower number of *de novo* introduced CpG dinucleotides than the E/NS1+176CpG variant—did not cause histopathology in mouse brains. While E/NS1+176CpG caused histopathology comparable to the wild-type variant (**Figure 2**). In accordance with these findings, we previously found that E+102CpG did not cause placental infection in pregnant mice, while E/NS1+176CpG was found in several placental samples (10). Our working hypothesis is that different extent of CpG enrichment in viral genomes may evoke different extent of self-damaging host immune responses in the brain; however, this hypothesis should be experimentally tested yet. While we demonstrated lesions using H&E staining, the limitation in the present study is that we did not test cellular and molecular mechanisms that determine phenotypical differences between CpG-enriched variants. In the future, viral protein expression of recoded variants and different cellular responses need to be studied in the brain. Together, our previous findings in the mouse pregnancy model (10) and current findings in the mouse brain suggest that infection phenotypes and safety of recoded vaccine candidates is not necessarily determined by the higher number of *de novo* introduced CpG dinucleotides. And each vaccine candidate would require a pathogen-specific CpG-recoding approach.

Recent Ebola and Zika virus epidemics—pathogens that have detrimental effects on mother and developing fetus—raised the critical need to evaluate vaccines in pregnant hosts (55). We have previously demonstrated the safety of CpG-recoded Zika virus vaccine candidates in the mouse pregnancy model (10). However, infection phenotypes caused by CpG-recoded variants in fetuses were unknown. This knowledge is important for understanding the outcomes of a hypothetical situation where recoded vaccine strains breach the placental barrier.

We directly inoculated several fetuses with the wild-type, E+102CpG, or E/NS1+176CpG Zika virus variant. After inoculation, the virus initially spreads from the index conceptuses to non-manipulated adjacent, and subsequently to more distant siblings providing an *in utero* transmission assay to compare the dynamics of productive trans-fetal infection between Zika virus strains or variants (24–26). CpG-recoded variants caused similar *in utero* infection kinetics as indicated by the efficient fetus-to-fetus virus spread and high viral loads in the placenta and amniotic fluids (**Figures 5A–D**). However, viral loads for the E/NS1+176CpG variant and particularly for the E+102CpG variant were considerably suppressed in fetal lymph nodes (**Figures 5E**, **F**). In general, lower viral loads in fetal lymph nodes than in the placenta (**Figures 5A, B** and **Figures 5E, F**) suggest that lymph nodes may play an important role in limiting Zika infection in fetuses. Further comparative studies in placental versus lymph node tissues may clarify fetal immune responses that efficiently restrict Zika infection. In support of different tissue tropism of virus variants with different CpG content, in the previous *in silico* study, the authors demonstrated an association between CpG content in the hantavirus genome, tissue tropism, and clinical outcomes in patients (56). The CpG content, however, was analyzed in non-coding genomic regions, and we do not know whether the same mechanisms are applied to variants with CpG-enriched content in non-coding and coding genomic regions.

Specifically to attenuated infection phenotypes caused by CpG-enriched viruses, it has been shown *in vitro* that ZAP recognizes and binds to CpG-enriched motifs in viral RNA; afterward, its antiviral activity is dependent on the interactions with other cellular proteins (8, 11, 12, 14, 15). Although the full complexity of ZAP cofactors involved in its antiviral activity is still unclear, there are several proteins that have been mechanistically validated: First, TRIM25, an interferon-stimulated gene protein, and E3 ubiquitin ligase have been identified to mediate ZAP ubiquitination, and complement ZAP to inhibit the replication of Sindbis virus, human cytomegalovirus, and HIV-1 containing elevated CpG dinucleotides (8, 14, 57, 58). TRIM25 interacts with ZAP through the SPRY domain, and downregulation of endogenous TRIM25 expression diminishes the antiviral functions of ZAP, suggesting that TRIM25 is required for ZAP antiviral activity (57, 59). Second, ZAP also interacts with cytoplasmic protein KHNYN to inhibit CpG-enriched viral RNA. In contrast to ZAP, which does not possess nuclease activity, KHNYN contains a KH-like domain and an NYN endonuclease domain. Knockdown of endogenous KHNYN reverts attenuation of CpG-enriched HIV-1. KHNYN depletion also increases MLV Gag expression and virion production in the presence of ZAP, suggesting that KHNYN is an essential ZAP cofactor for antiviral activity against CpG-enriched HIV-1 (15). Third, a recent report identified OAS3/RNase L pathway complementation with ZAP in restricting the replication of echovirus 7 enriched with UpA and CpG dinucleotides. Depletion of Ribonuclease L (RNase L) and oligoadenylate synthase 3 (OAS3) recovers the replication of CpG-high and UpA-high echovirus 7 variants in the presence of abundant ZAP expression suggesting the dependence on the expression of ZAP, OAS3, and RNAseL for CpG/UpA-mediated attenuation (11). In addition, several components of the 5′−3′ and 3′−5′ RNA degradation pathways have been proposed to interact with ZAP to target viral RNA for degradation, such as CP1A-DCP2, XRN1, PARN, and the exoribonuclease exosome complex. Knockdown of these cytoplasmic proteins abrogates the antiviral activity of ZAP (60–62). Mass spectrometry interactome studies identified multiple proteins directly interacting and co-localizing with ZAP (61). Identifying the full spectrum of ZAP cofactors and mechanisms of how they act will be critical to elucidate the ZAP antiviral system and develop better CpG-recoded live vaccines. Comparative studies in placental versus lymph node tissues may provide an experimental tool for a better functional understanding of known immune mechanisms that restrict infection caused by CpG-enriched viruses *in vitro*, and identification of new mechanisms of CpG-recoded virus-host interactions *in vivo*. For example, it will be interesting to compare expression of different ZAP isoforms and their known interactions with RIG-I-mediated induction of type I interferons (63) in fetal lymph nodes with restriction of CpG-enriched Zika viruses (**Figures 5E**, **F**) and in the placenta with no restriction (**Figures 5A**, **B**).

Like with histology findings in adult mouse brains (**Figure 2**), findings in lymph nodes with lower viral loads of the ZIKV E+102CpG variant—the variant with the lower number of *de novo* introduced CpG dinucleotides than the E/NS1+176CpG variant—are counterintuitive. Again suggesting that infection phenotypes and the safety of recoded vaccine candidates are not exclusively determined by the number of *de novo* introduced CpG dinucleotides.

We showed that both wild-type and CpG-enriched variants can persist in fetal placental mesenchyme for at least 28 days (**Figures 5A**, **B**). These data suggest that the major risks of vaccination with CpG-recoded vaccines during pregnancy are associated with potential transmission of a vaccine variant from mother to fetus and subsequent virus persistence in the fetal part of the placenta—fetal placental mesenchyme. Based on our study in pregnant mice where maternal immunity efficiently prevents the transplacental spread of CpG-recoded Zika virus variants (10), it is unlikely that recoded vaccine variants will replicate in maternal tissues of other animal species with titers sufficient to cause transplacental spread to the fetal part of the placenta. But whether recoded viruses will show the same suppression of maternal infection in pregnant non-human primates and humans as in mice is yet unknown. Supporting potential application, vaccination with Yellow fever vaccine during human pregnancy did not show safety concerns in a retrospective study (64). A combination of CpG recoding with other established live vaccine strategies should be also tested to mitigate potential transplacental infection. For example, CpG-recoded vaccine candidates with inserted targets for placenta-specific microRNAs (65) may provide safer recoded vaccines.

Depletion of CpG dinucleotides in genomes of wild viruses from field samples has been linked to virus evolution, host-switching, pathogenicity, and innate immune responses (66, 67). However, depletion of CpG dinucleotides in genomes of synthetic recoded viruses during *in vivo* infection is not well studied. Comparative evolutionary studies of wild-type and CpG-recoded virus variants *in vivo* are challenging because after peripheral inoculation, CpG-recoded viruses show attenuated infection phenotypes with no or low viral loads (3, 10). Having comparable RNA loads of wild-type and CpG-recoded Zika virus in mouse brains and porcine placenta after direct intracerebral and *in utero* inoculations, we compared the evolution of different variants.

Most *de novo* introduced CpG dinucleotides were preserved in sequences of recoded Zika virus variants during 7-day infection in mouse brains and 28-day infection in the porcine placenta showing the stability of recoded viruses. However, *in vivo* evolution resulted in comparable mutation frequencies and entropy in wild-type and recoded variants (**Figures 3**, **8**), questioning whether emerging mutations may affect vaccine virus phenotypes. While each ZAP molecule binds one CpG dinucleotide, multiple ZAP molecules may form an oligomer on a target RNA, and recoded vaccine candidates with hundreds of extra CpG dinucleotides most probably will show rare or no reversion to virulence, additional studies are needed to identify whether the emergence of mutations which do not affect CpG dinucleotides within recoded, adjacent, or distant viral genomic regions may affect the safety of recoded vaccines.

The relatively small number of SNVs in samples from individual animals did not allow us to accurately analyze selective pressure in different virus variants. The duration of Zika evolution in mouse brains was only seven days after inoculation, when virus loads in the brain were sufficient to construct libraries for NGS. Animal models supporting more prolonged infection in the brain or serial *in vivo* passages are needed to better understand the comparative evolution of wild-type and CpG-enriched variants. In the porcine placenta where Zika caused longer persistent infection for 28 days after inoculation, five SNVs in the wild-type virus and only one SNV in the CpG-enriched variant reached nearly 100% frequency, suggesting positive selection and potential fixation of these SNVs in the virus population. However, to confirm stronger positive selection and more efficient fixation of SNVs in the wild-type virus variants than in CpG-enriched variants, prolonged *in vivo* passages are needed.

It is suggested that CpG dinucleotide depletion in viral genomes is an evolutionary strategy to avoid host antiviral responses which specifically target high genomic CpG content (8). Our *in vivo* data suggest that evolutionary pressures on the virus genomic CpG content can vary in different organs—i.e., higher in fetal lymph nodes where infection was more efficiently suppressed (**Figures 5E**, **F**), and lower in the placenta where infection was less efficiently suppressed (**Figures 5A**, **B**), advocating the different evolutionary roles of different host tissues in historical depletion of viral CpG dinucleotides. Prolonged serial *in utero* passaging of virus variants with different CpG content and whole-genome NGS may partially test this hypothesis.

To summarize, we used well-established mouse and porcine models to study infection phenotypes of the CpG-enriched neurotropic and congenital virus—Zika virus, directly in the target tissues—the brain and placenta. We showed that wild-type and CpG-recoded Zika virus variants had similar infection kinetics in mouse brains after direct intracerebral injection, and in the placenta after direct *in utero* injection. However, while overall infection kinetics were comparable between wild-type and recoded variants, we found convergent phenotypical differences characterized by reduced pathology in the mouse brain and reduced replication of CpG variants in fetal lymph nodes. Our study highlighted potential safety risks of CpG-recoded live vaccines and emphasized further directions to fine-tune the CpG recoding vaccine approach for better safety. Also, our data may inform future immunization strategies with restrictions in pregnant women and individuals with immunodeficiencies.

## Data availability statement

The data presented in the study are deposited in the Sequence Read Archive (SRA) repository, BioProject accession number PRJNA837685.

## Ethics statement

We conducted animal experiments following the Canadian Council on Animal Care standards for the ethical use and care of animals in science. Animal studies were approved by the University of Saskatchewan’s Animal Research Ethics Board (#20180012). All efforts were made to minimize animal suffering. Mice were euthanized by isoflurane and exsanguination *via* cardiac puncture. To ensure death, the chest cavity was opened during tissue collection. Pigs were euthanized with an anesthetic overdose and exsanguination.

## Author contributions

Conceptualization, project administration, and supervision: UK. Data curation: DU and UK. Formal analysis and validation: DU, IT, and UK. Funding acquisition: DU and UK. Resources: UK. Investigation: DU, IT, NB, and UK. Methodology, software, visualization, and writing—original draft preparation: DU, IT, and UK. Writing—review and editing: DU, IT, NB, and UK. All authors contributed to the article and approved the submitted version.

## Funding

This work was supported by grants #420249 and #421645 to UK from Natural Sciences and Engineering Research Council (NSERC). DU received a Scholarship from the School of Public Health, University of Saskatchewan. VIDO receives operational funding from the Government of Saskatchewan through Innovation Saskatchewan and the Ministry of Agriculture and from the Canada Foundation for Innovation through the Major Science Initiatives for its CL3 facility (InterVac). The funders had no role in study design, data collection and analysis, decision to publish, or preparation of the manuscript.

## Acknowledgements

We thank VIDO animal care technicians and veterinarians for help with animal experiments. Published as VIDO manuscript #970.

## Conflict of interest

The authors declare that the research was conducted in the absence of any commercial or financial relationships that could be construed as a potential conflict of interest.

## Publisher’s note

All claims expressed in this article are solely those of the authors and do not necessarily represent those of their affiliated organizations, or those of the publisher, the editors and the reviewers. Any product that may be evaluated in this article, or claim that may be made by its manufacturer, is not guaranteed or endorsed by the publisher.
