# Supplementary material for: CpG content in the Zika virus genome affects infection phenotypes in the adult brain and fetal lymph nodes
Source: Front Immunol. 2022 Aug 2;13:943481. doi: 10.3389/fimmu.2022.943481 (PMC9379343; doi:10.3389/fimmu.2022.943481)

ZIKV-WT-stock, replicate a

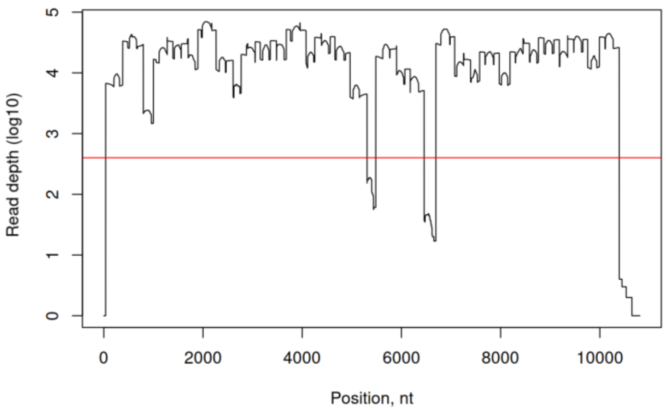

ZIKV-WT-stock, replicate b

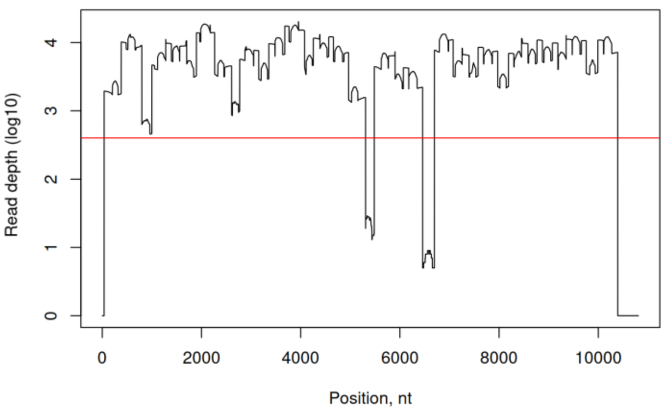

313-10-F9, replicate a

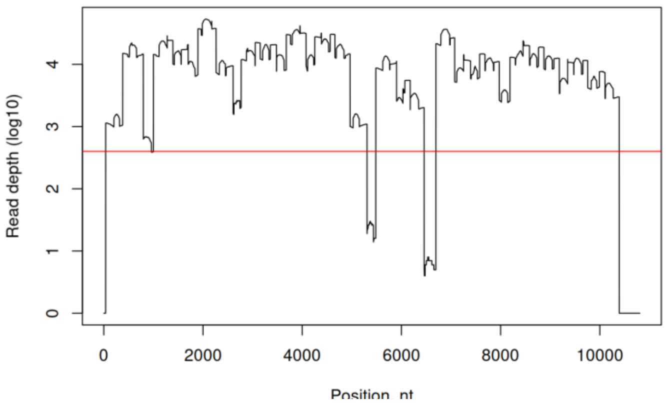

313-10-F9, replicate b

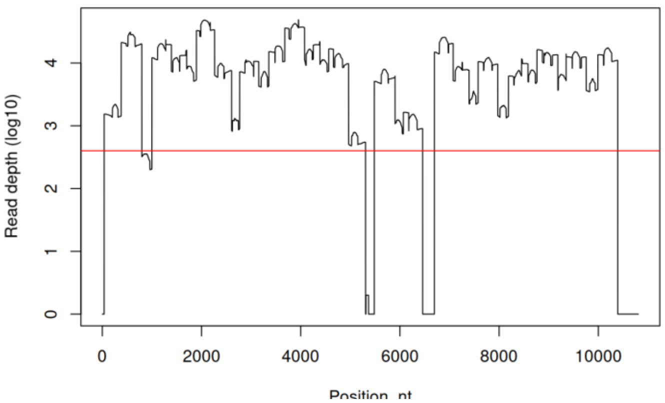

313-10-F11, replicate a

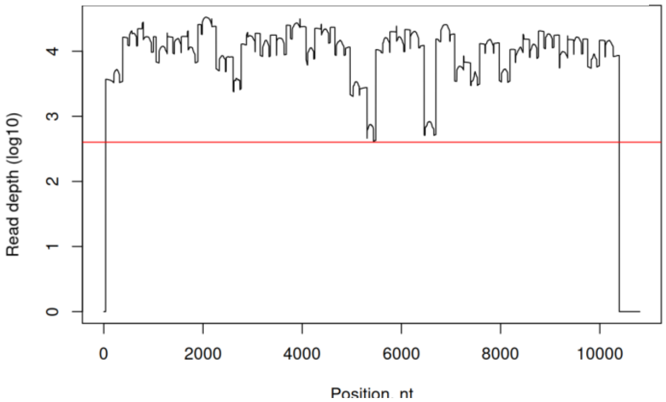

313-10-F11, replicate b

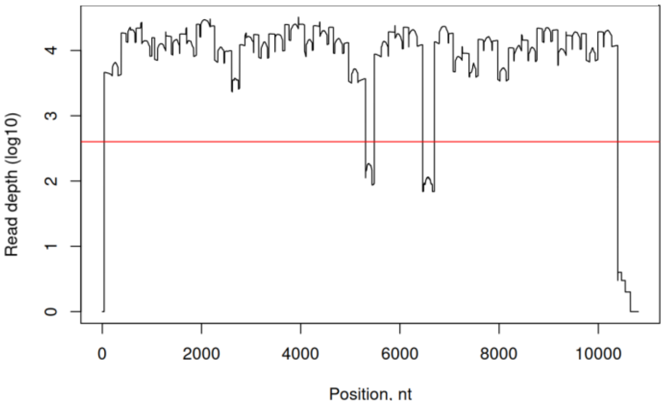

313-10-F12, replicate a

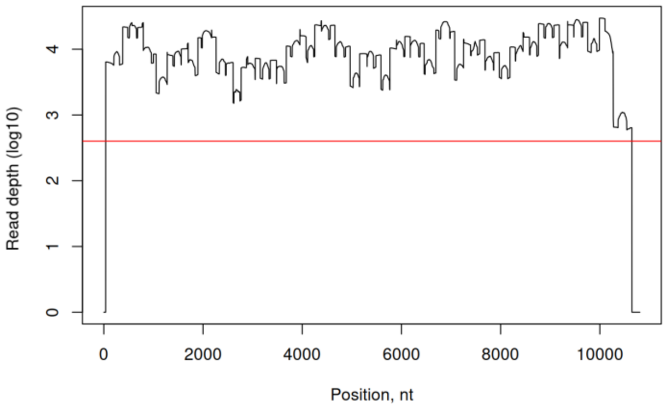

313-10-F12, replicate b

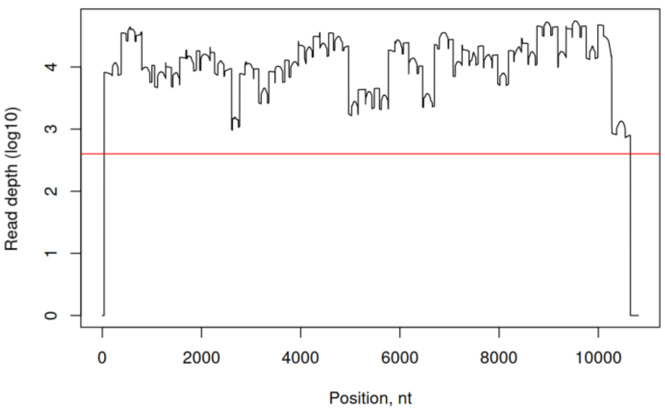

313-10-F15, replicate a

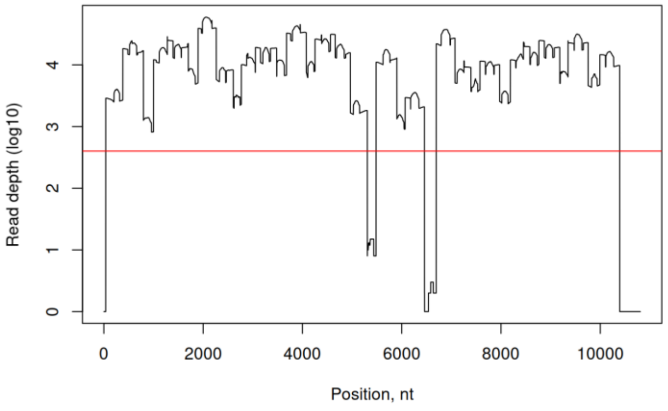

313-10-F15, replicate b

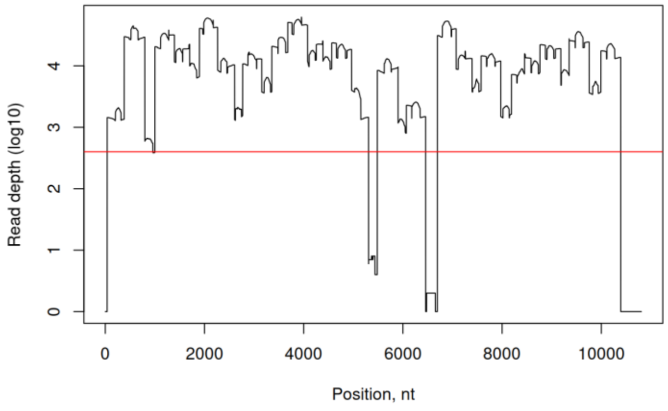

ZIKV-E/NS1+176CpG-0p, replicate a

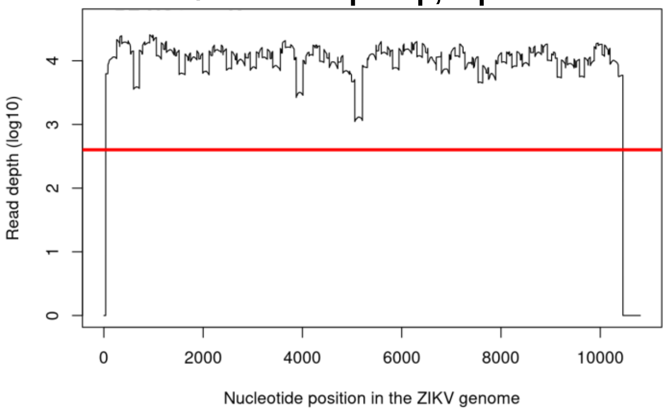

ZIKV-E/NS1+176CpG-0p, replicate b

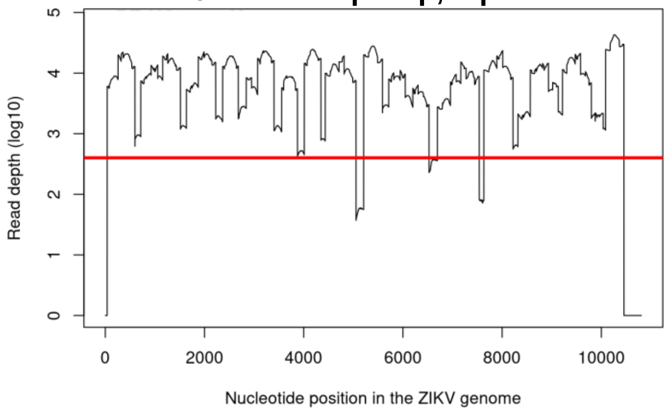

313-8-F12, replicate a

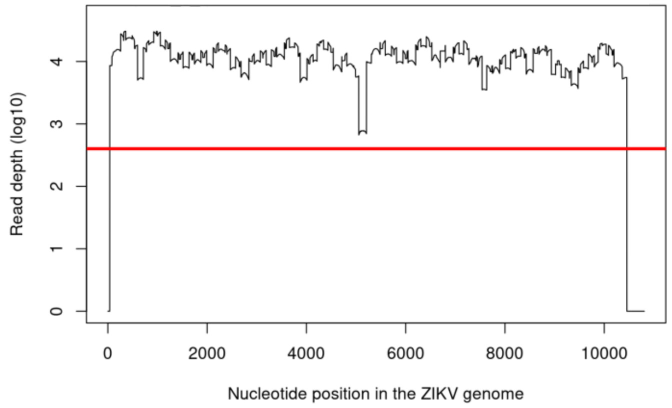

313-8-F12, replicate b

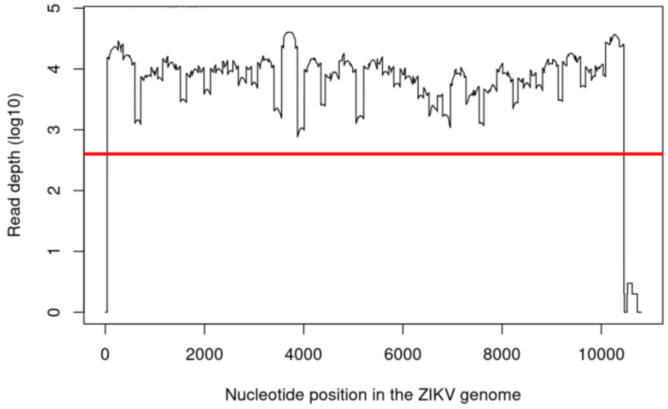

313-8-F13, replicate a

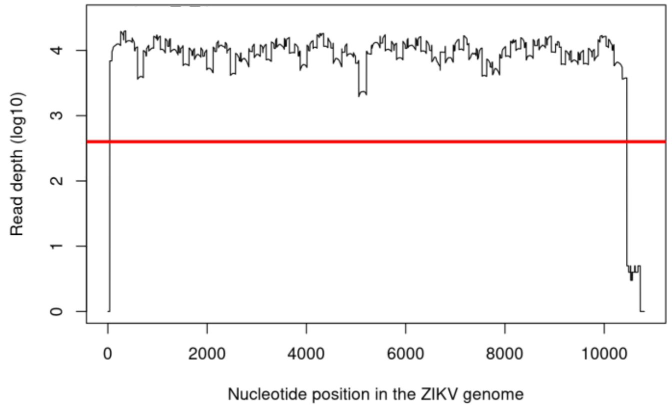

313-8-F13, replicate b

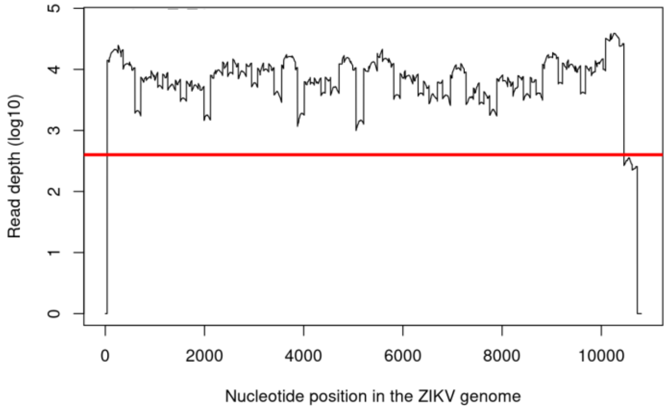

313-8-F14, replicate a

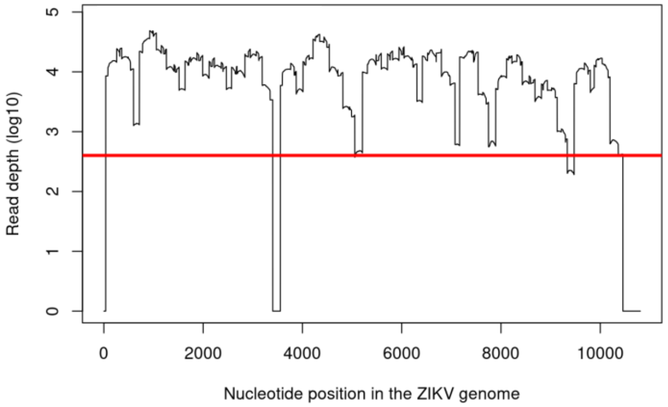

313-8-F14, replicate b

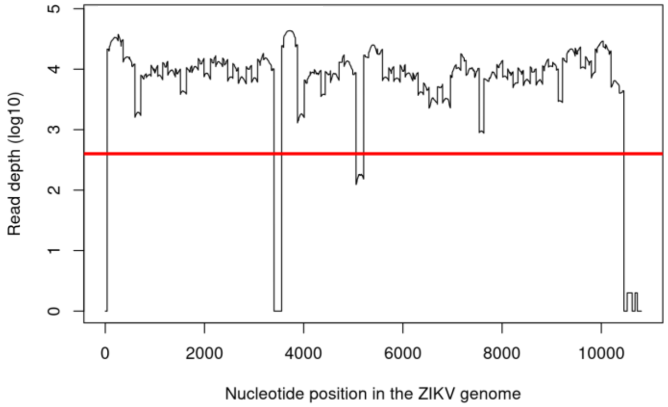

313-8-F15, replicate a

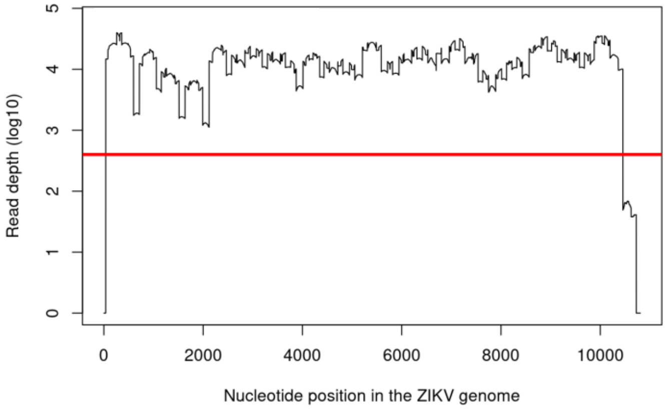

313-8-F15, replicate b

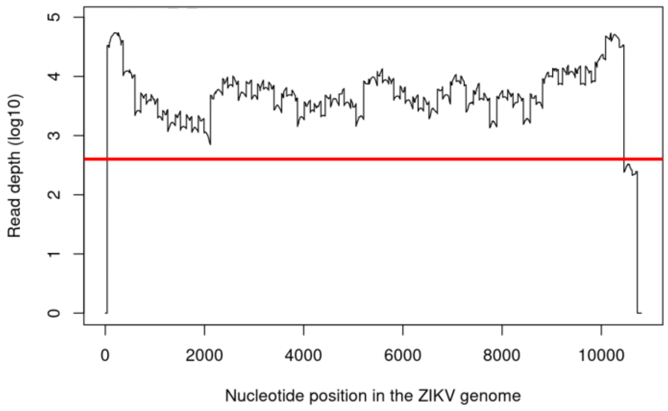

Supplement: Supplementary file 1 [file DataSheet_1.zip › Data Sheet 1 (1)/Supplementary/Supplementary File 3 Zika virus NGS coverage in the porcine placenta.pdf]
