# Supplementary material for: CpG content in the Zika virus genome affects infection phenotypes in the adult brain and fetal lymph nodes
Source: Front Immunol. 2022 Aug 2;13:943481. doi: 10.3389/fimmu.2022.943481 (PMC9379343; doi:10.3389/fimmu.2022.943481)
Supplement: Supplementary file 1 [file DataSheet_1.zip › Data Sheet 1 (1)/Supplementary/Supplementary Table 1 Bioplex reagents.pdf]

Table S1 Bio-Plex reagents

Cytokine	Capture antibody and Supplier	Detection antibody, Supplier, and Final Concentration	Standard, Supplier, and Final Concentration	Bead and Supplier
**IL-1*β***	MAb anti porcine IL-1β/IF2; R&D MAB6811	Goat anti porcine IL-1*β*/IF2 biotin; R&D BAF681; 0.5 μg/ml	Recombinant porcine IL-1*β*/IF2; R&D 681-PI-10; 5000 pg/ml	Region 26; BioRad MC10026-01
**IL-6**	Goat anti porcine IL-6; R&D AF686	Goat anti porcine IL-6 biotin; R&D BAF686; 0.5 μg/mL	Recombinant porcine IL-6; R&D 686-PI-025; 5000 pg/mL	Region 65; BioRad MC10065-01
**IL-8**	MAb anti sheep IL8 (86.9% homology); AbD Serotec MCA1660	MAb anti porcine CXCL8/IL8 biotin; R&D MAB5351; biotinylated in house; 1 μg/mL	Recombinant porcine IL-8; Kingfisher RP0109S-005; 200 pg/ml	Region 27; BioRad MC10027-01
**IL-10**	MAb anti swine IL-10; Invitrogen ASC0104	MAb anti swine IL-10 biotin; Invitrogen ASC9109; 0.5 μg/mL	Recombinant swine IL-10; Invitrogen PSC0104; 5000 pg/mL	Region 28; BioRad MC10028-01
**IL-12**	MAb anti porcine IL-12; Kingfisher MA0413S-100	MAb anti porcine IL12/IL23 p40 biotin; R&D BAM9122; 0.5 μg/mL	Recombinant porcine IL-12; R&D 912-PL-025; 5000 pg/mL	Region 36; BioRad MC10036-01
**IL-13**	Goat anti swine IL-13; Kingfisher PB0094S-100	Goat anti swine IL-13 biotin; Kingfisher PBB0096S-050; 0.5 μg/ml	Recombinant swine IL-13; Kingfisher RP0007S-005; 5000 pg/ml	Region 52; BioRad MC10052-01
**IL-17A**	Rabbit anti porcine IL-17A; Kingfisher KP0498S-100	Rabbit anti porcine IL-17A biotin; Kingfisher KPB0499S-050; 0.1 μg/mL	Recombinant porcine IL-17A; Kingfisher RP0128S-005; 2000 pg/mL	Region 62; BioRad MC10062-01
**IFN-γ**	MAb anti porcine IFN-γ; Fisher ENMP700	MAb anti porcine IFN-γ; Fisher ENPP700; biotinylated in-house; 0.4 μg/mL	Recombinant porcine IFN-γ; Ceiba Geigy; 2000 pg/ml	Region 43; BioRad MC10043-01
**IFN-α**	MAb anti porcine IFN-α; GeneTex GTX11408	MAb anti pig IFN-α; PBL 27105-1; biotinylated in house; 0.1 μg/mL	Recombinant porcine IFNα; Genentech; 200 pg/mL	Region 45; BioRad MC10045-01
