# Supplementary figures and images for: CpG content in the Zika virus genome affects infection phenotypes in the adult brain and fetal lymph nodes
Source: Front Immunol. 2022 Aug 2;13:943481. doi: 10.3389/fimmu.2022.943481 (PMC9379343; doi:10.3389/fimmu.2022.943481)

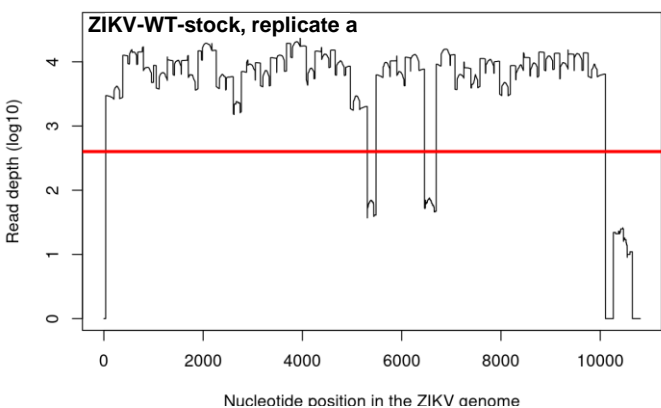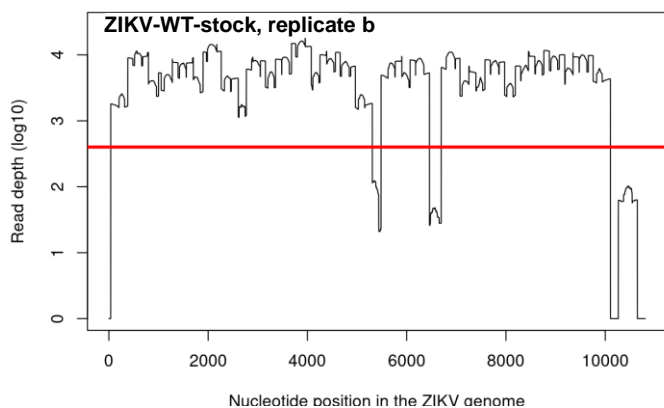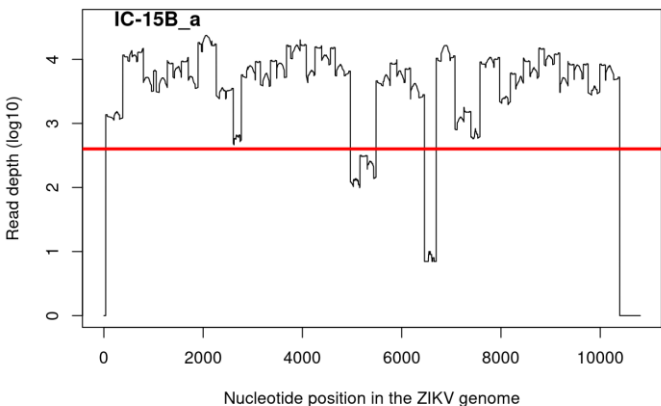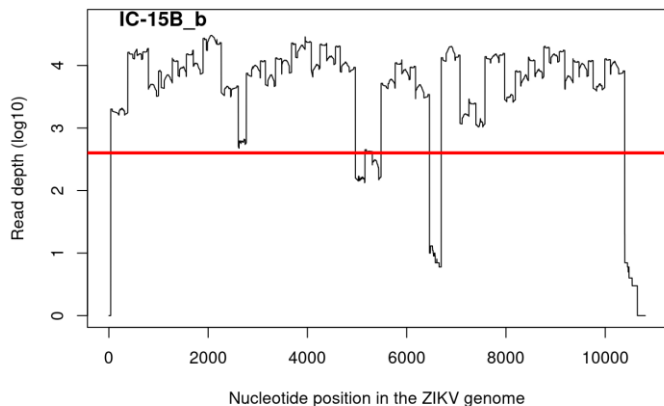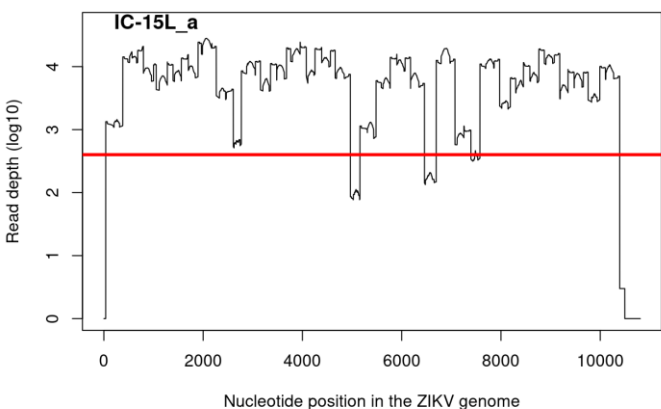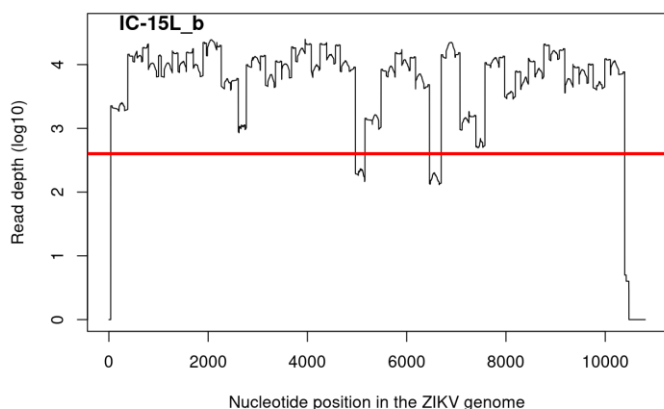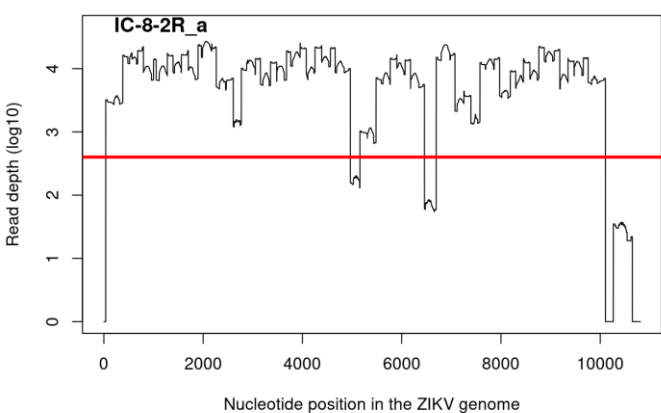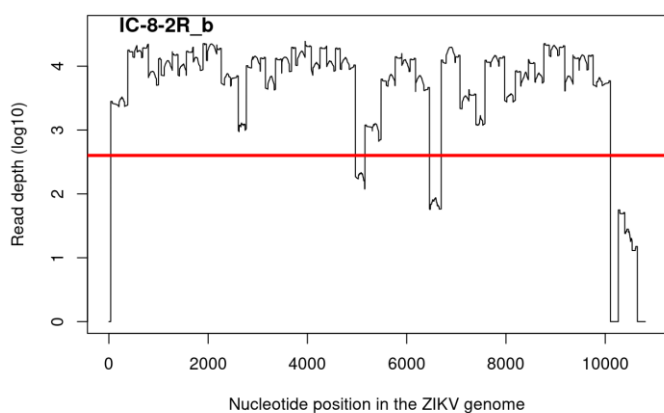

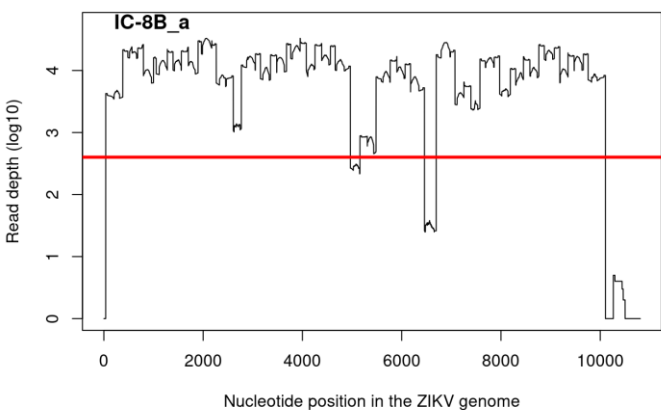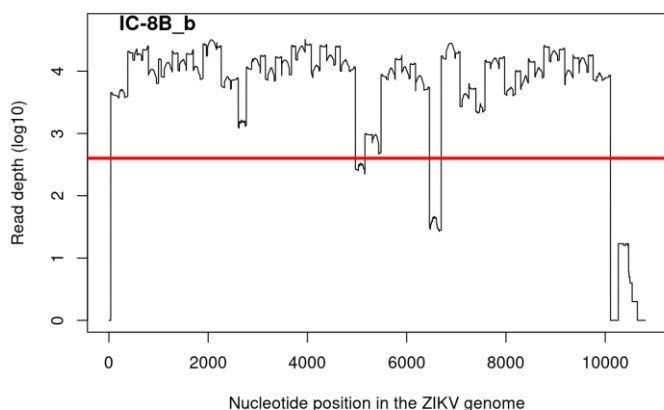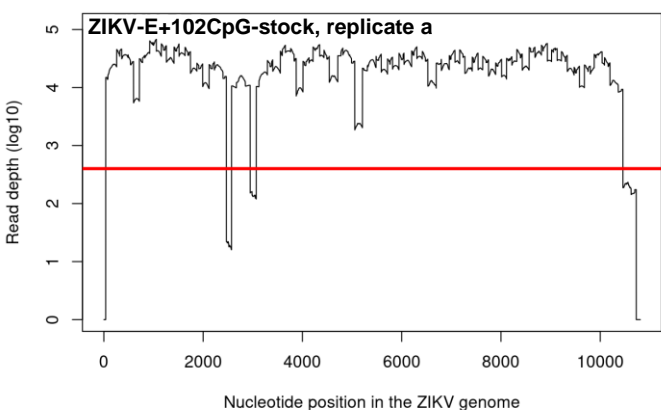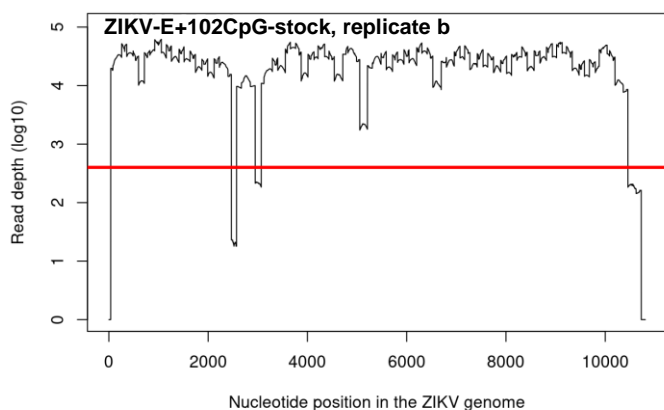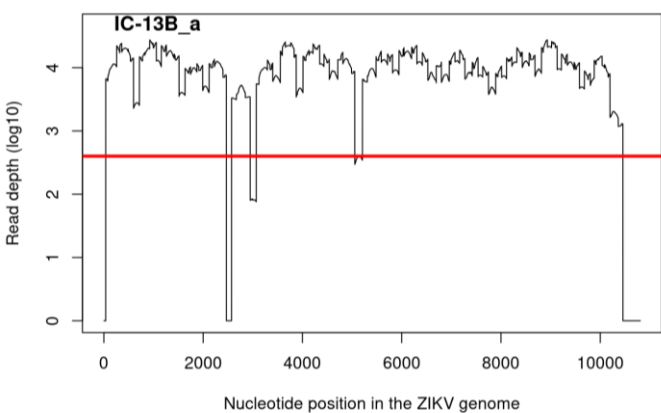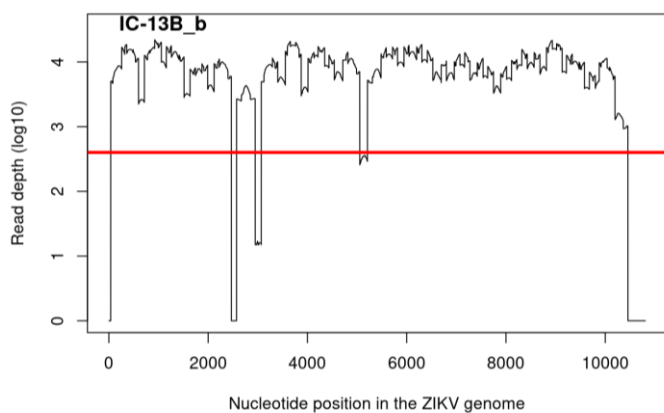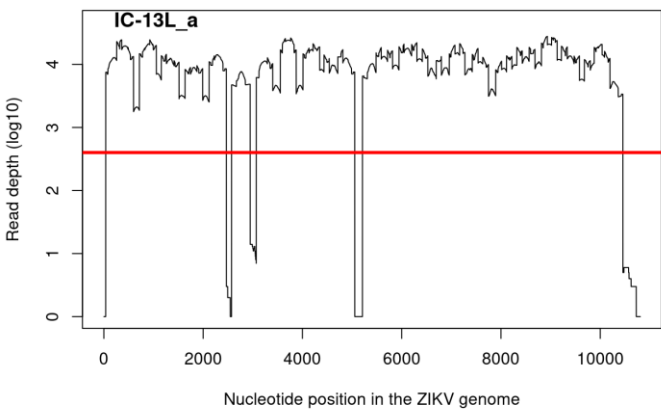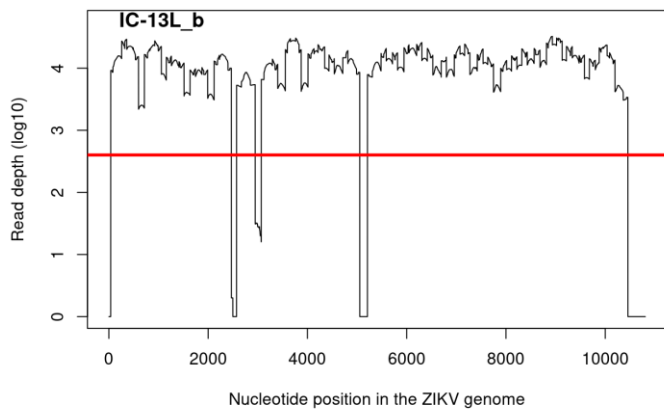

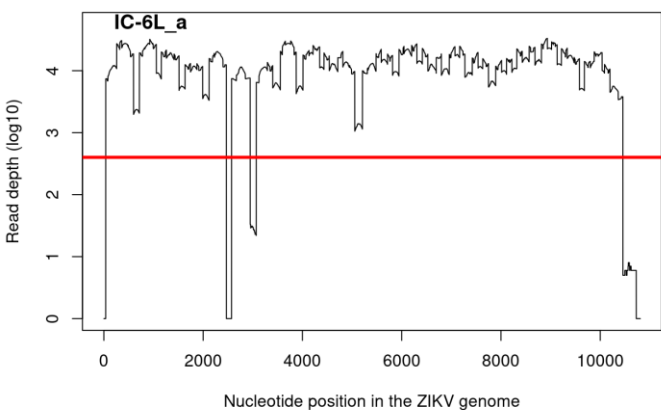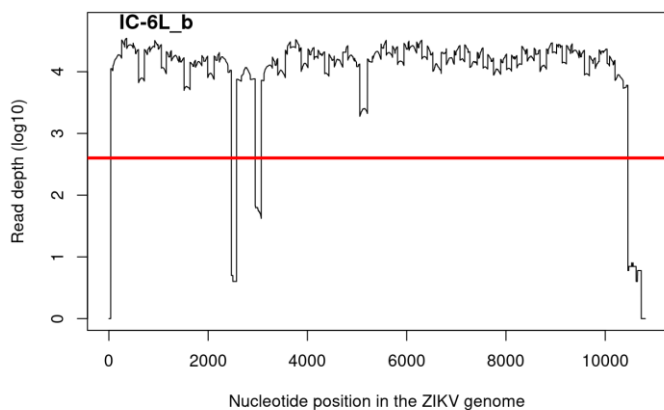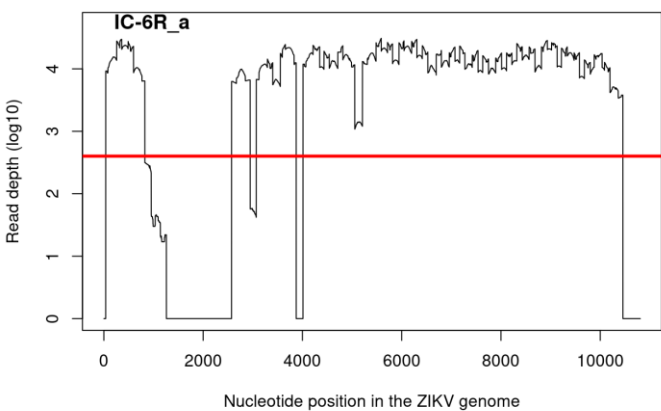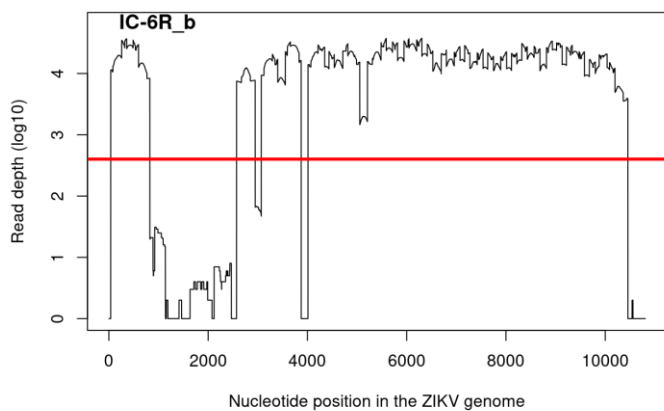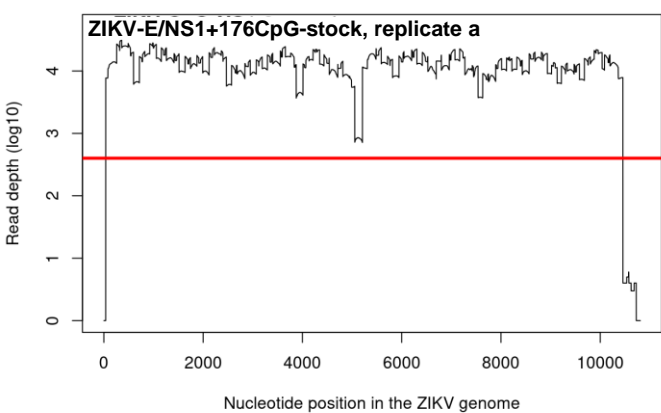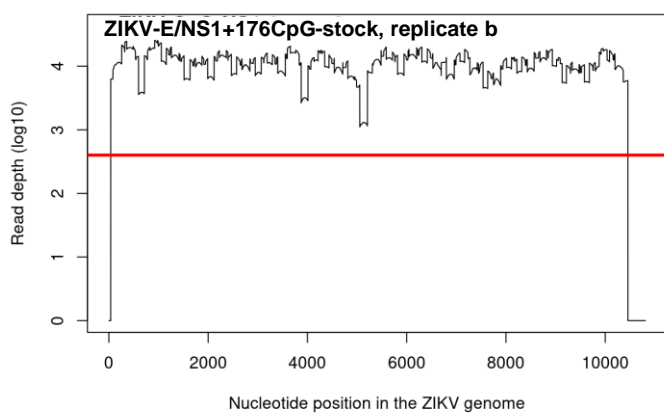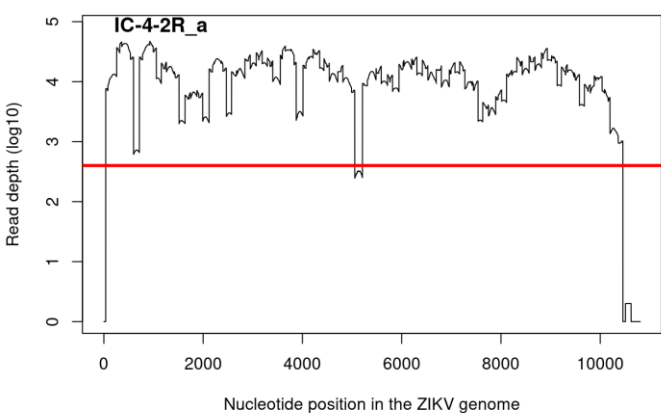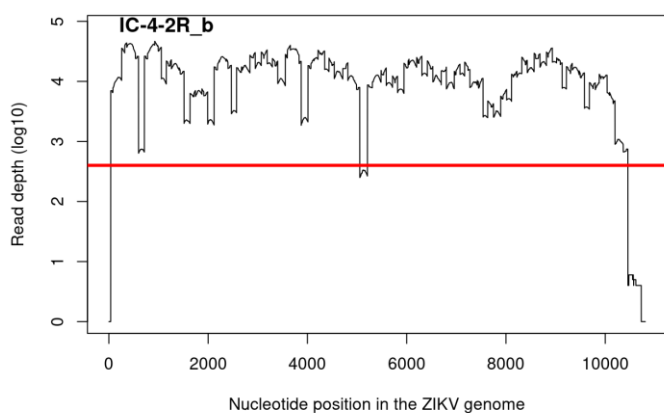

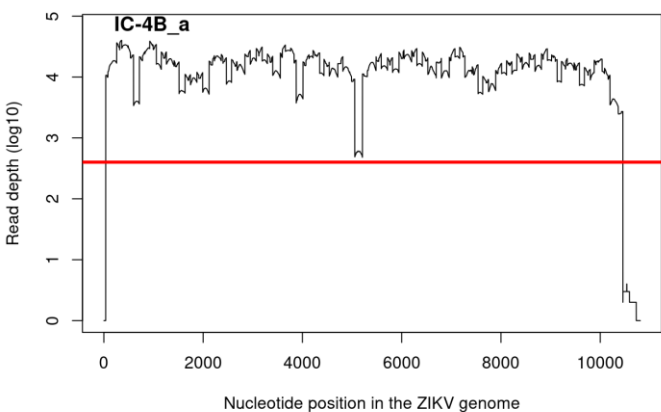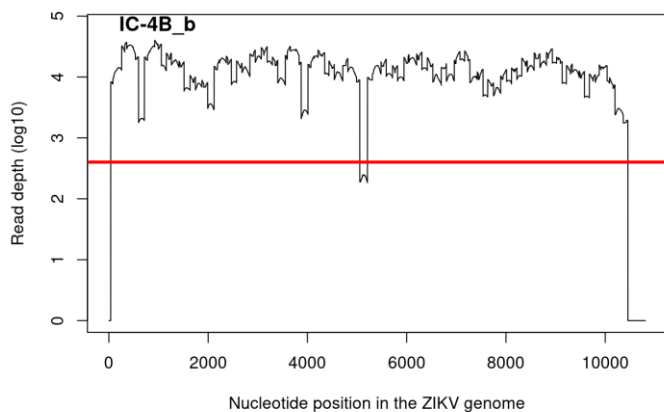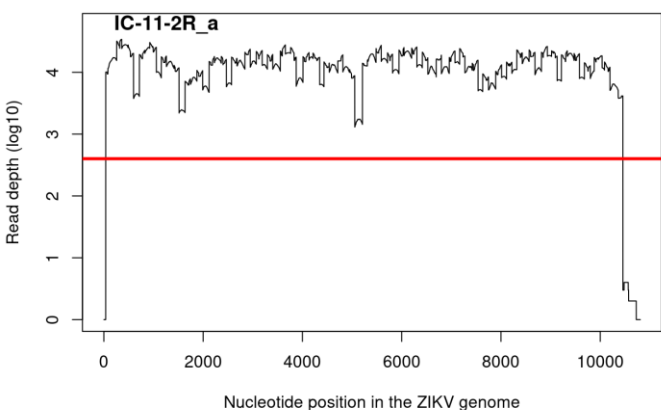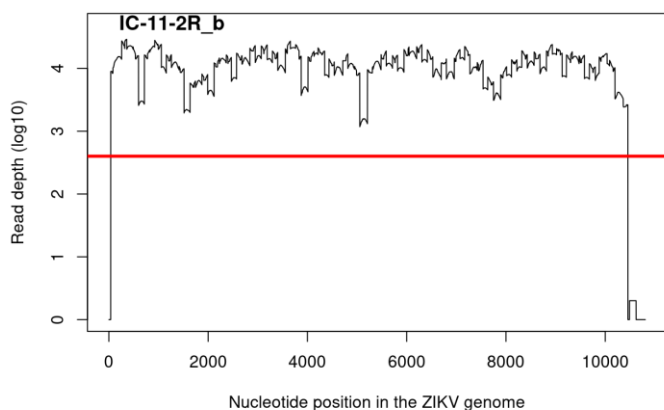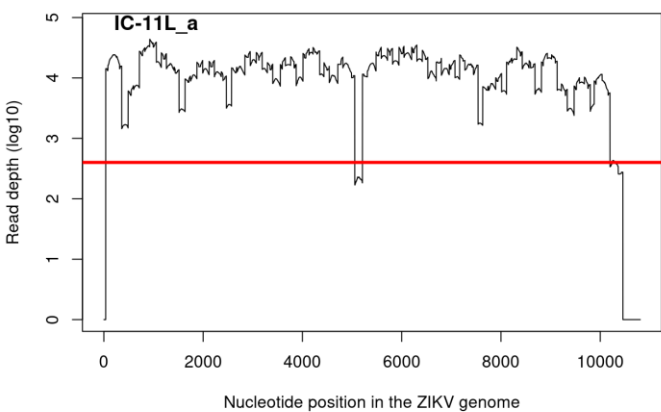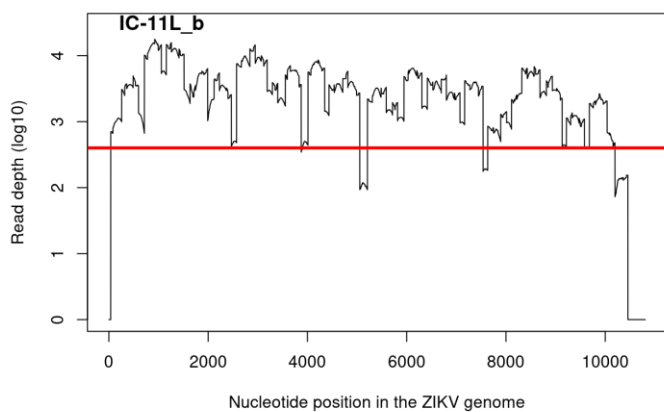

Supplement: Supplementary file 1 [file DataSheet_1.zip › Data Sheet 1 (1)/Supplementary/Supplementary File 2 Zika virus NGS coverage in the mouse brain sampels.pdf]
